# ICOS costimulation in combination with CTLA-4 blockade remodels tumor-associated macrophages toward an antitumor phenotype

**DOI:** 10.1084/jem.20231263

**Published:** 2024-03-22

**Authors:** Naveen Sharma, Xiaozhou Fan, Oluwatomisin T. Atolagbe, Zhongqi Ge, Kelly N. Dao, Padmanee Sharma, James P. Allison

**Affiliations:** 1 Department of Immunology, The University of Texas MD Anderson Cancer Center, Houston, TX, USA; 2 James P. Allison Institute, The University of Texas MD Anderson Cancer Center, Houston, TX, USA; 3 Immunotherapy Platform, James P. Allison Institute, The University of Texas MD Anderson Cancer Center, Houston, TX, USA; 4 Parker Institute for Cancer Immunotherapy, The University of Texas MD Anderson Cancer Center, Houston, TX, USA; 5 Department of Genitourinary Medical Oncology, The University of Texas MD Anderson Cancer Center, Houston, TX, USA

## Abstract

We have previously demonstrated synergy between ICOS costimulation (IVAX; ICOSL-transduced B16-F10 cellular vaccine) and CTLA-4 blockade in antitumor therapy. In this study, we employed CyTOF and single-cell RNA sequencing and observed significant remodeling of the lymphoid and myeloid compartments in combination therapy. Compared with anti-CTLA-4 monotherapy, the combination therapy enriched Th1 CD4 T cells, effector CD8 T cells, and M1-like antitumor proinflammatory macrophages. These macrophages were critical to the therapeutic efficacy of anti-CTLA-4 combined with IVAX or anti-PD-1. Macrophage depletion with clodronate reduced the tumor-infiltrating effector CD4 and CD8 T cells, impairing their antitumor functions. Furthermore, the recruitment and polarization of M1-like macrophages required IFN-γ. Therefore, in this study, we show that there is a positive feedback loop between intratumoral effector T cells and tumor-associated macrophages (TAMs), in which the IFN-γ produced by the T cells polarizes the TAMs into M1-like phenotype, and the TAMs, in turn, reshape the tumor microenvironment to facilitate T cell infiltration, immune function, and tumor rejection.

## Teaser

The study unveils that potent combination therapies remodel TAMs to antitumor proinflammatory M1-like macrophages crucial for their therapeutic efficacy. It demonstrates a positive feedback loop between intratumoral effector T cells and TAMs, mediated by IFN-γ reshaping the tumor microenvironment.

## Introduction

In recent years, immunotherapy has won wide acceptance as an effective therapeutic option for cancers with its potential to achieve complete response and durable immune memory. The Food and Drug Administration’s 2011 approval of ipilimumab, an anti-CTLA-4 antibody, marked a milestone for advanced melanoma patients, demonstrating extended survival in a Phase III trial (Hodi et al., 2010; Robert et al., 2011). Drugs targeting other immune checkpoints have since been tested, especially anti-PD-1/PD-L1, and exhibited promising clinical results across cancer types (Zou et al., 2016). However, these checkpoint blockade drugs’ objective response rate and overall survival indicate untapped potential.

A member of the CD28/CTLA-4 family, the inducible T cell costimulator (ICOS), is a T cell–specific protein shown to enhance the efficacy of immune checkpoint blockade therapy (Fan et al., 2014; Hutloff et al., 1999; Sharpe and Freeman, 2002). Activated T cells upregulate ICOS, and in the context of CTLA-4 blockade, a novel Th1 cell population expressing ICOS emerges that expresses T-bet, IFN-γ, and PD-1 (Liakou et al., 2008; Wei et al., 2017). ICOS^+^ Th1-like cells play a crucial role in the antitumor effect of anti-CTLA-4 therapy, constituting the majority of tumor-specific, IFN-γ producing CD4 T cells (Carthon et al., 2010; Liakou et al., 2008; Vonderheide et al., 2010). Our earlier work demonstrated a correlation between persistent elevation of ICOS^+^ CD4 T cells in the peripheral blood after ipilimumab therapy and improved survival (Carthon et al., 2010). Not only is it a pharmacodynamic biomarker to assess the response to anti-CTLA-4 treatment, but ICOS can also be targeted in combination with CTLA-4 blockade for enhanced tumor rejection, substantiated by evidence from ICOS/ICOSL pathway–deficient mice showing a weakened antitumor response compared to wild-type mice after anti-CTLA-4 treatment (Fu et al., 2011).

In our previous study, we activated the ICOS pathway using an ICOSL-transduced cellular vaccine (IVAX), significantly enhancing anti-CTLA-4 monotherapy’s efficacy by relying on a robust type 1 T cell–mediated response (Fan et al., 2014). However, CD8 T cells only partially contributed to tumor rejection. Tumor-associated macrophages (TAMs), a major tumor-infiltrating cell population, play diverse roles in cancer prognosis. While often linked to poor outcomes, some cancers positively correlate with TAMs (Bingle et al., 2002; Kim et al., 2008; Zhang et al., 2012). The varied phenotypes and functions of macrophages may explain this apparent contradiction. Macrophages are traditionally categorized as M1- and M2-like; M1-like macrophages display potent antimicrobial and antitumor activity, while M2-like macrophages participate in parasite containment, tissue remodeling, and tumor progression (Mantovani et al., 2002). Notably, M1 and M2 represent opposite ends of a spectrum of functional states that macrophages can adopt. In this study, we used high-dimensional profiling techniques such as single-cell RNA sequencing (scRNA-seq) and mass cytometry (cytometry by time of flight [CyTOF]) to analyze changes in tumor-infiltrating immune cell populations. Our data suggest that TAMs play an essential role in combination therapy efficacy, with ICOS costimulation and anti-CTLA-4 blockade therapy profoundly remodeling both lymphoid and myeloid compartments.

## Results

### Macrophages are essential to the therapeutic efficacy of anti-CTLA-4 and IVAX combination therapy

**Figure S1.**
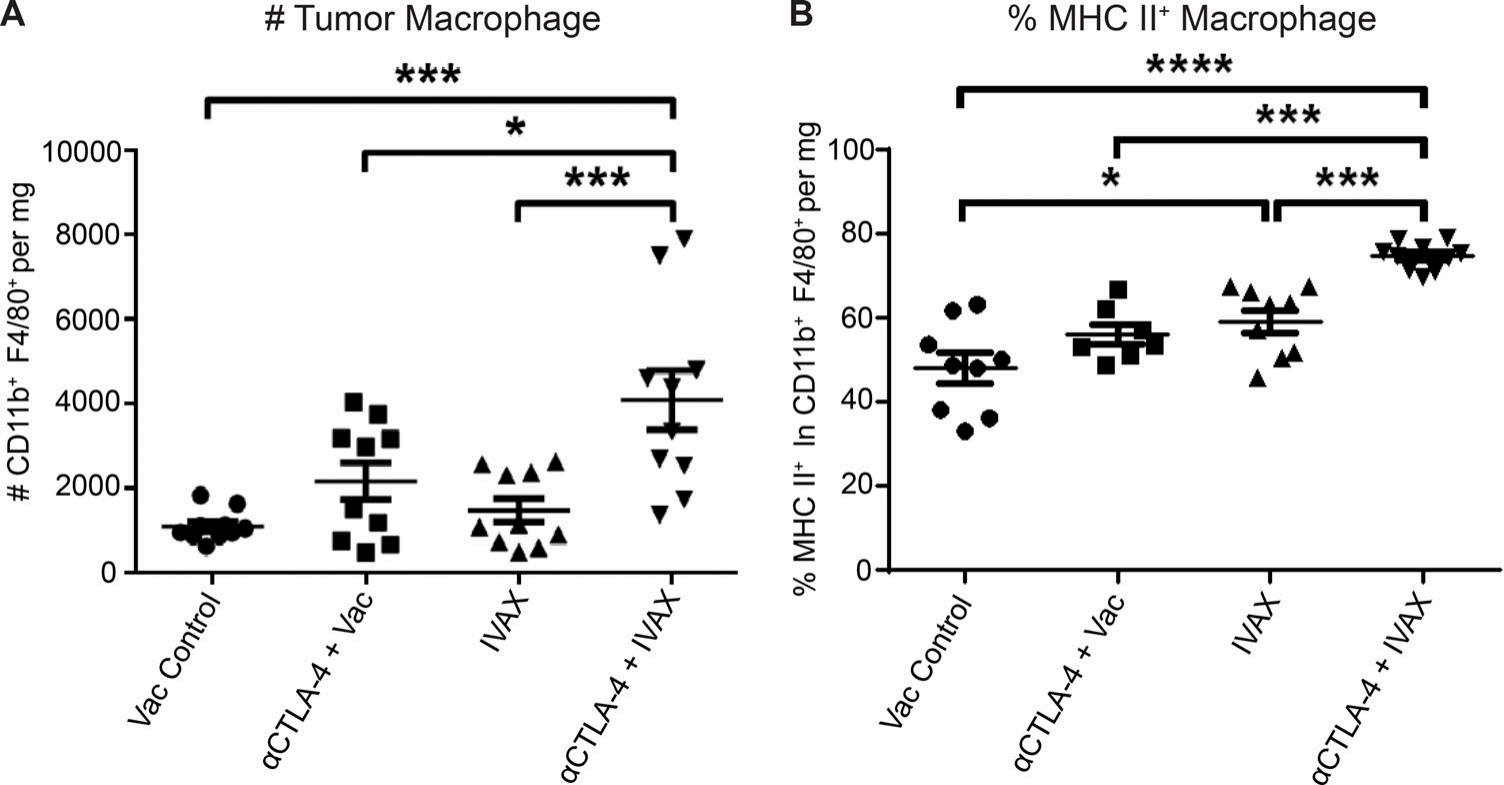
**A combination of IVAX and CTLA-4 blockade increases macrophage infiltration. (A)** The density of CD11b^+^ F4/80^+^ TAMs is depicted as an absolute number of cells per mg of tumor on day 16 after tumor challenge. The number of cells in tumors was calculated as described in the Materials and methods. Data are pooled from two independent experiments (*n* = 5 mice per group; one-way ANOVA, post hoc, *P < 0.05; ***P < 0.001). **(B)** Frequency of MHC class II molecule expressed on the surface of TAMs. Data are pooled from two independent experiments (*n* = 3–5 mice per group; one-way ANOVA, post hoc, *P < 0.05; ***P < 0.001; ****P < 0.0001). Error bars represent means ± SEM. Irradiated parental B16 tumor cells (Vac) were used as a control for the IVAX group.

In prior studies, we demonstrated synergistic increased tumor protection with the concurrent activation of the ICOS pathway and CTLA-4 blockade in mouse melanoma and prostate cancer models (Fan et al., 2014). While IFN-γ was crucial for optimal tumor protection, CD8 T cells played a partial role. This observation prompted us to examine the roles of other effector immune cell populations in the tumor microenvironment (TME). Compared with tumors treated with CTLA-4 blockade monotherapy, tumors treated with the combination of CTLA-4 blockade and IVAX were infiltrated with more CD11b^+^ F4/80^+^ macrophages (Fig. S1 A). Although TAMs have been extensively characterized as one of the significant immunosuppressive components of the TME, most of the macrophages infiltrating tumors treated with the combination therapy expressed high levels of MHC class II molecule, a marker of type 1 proinflammatory macrophages (Fig. S1 B). This suggests a deviation from conventional protumor TAMs.

To test the hypothesis that macrophages contribute to the antitumor efficacy of IVAX plus anti-CTLA-4 combination therapy, we sought to deplete macrophages within this treatment. Clodronate liposomes and anti-colony-stimulating factor 1 (CSF1) or colony-stimulating factor 1 receptor (CSF1R) antibodies have been used widely as a method for depleting macrophages in literature (van Rooijen et al., 1996; Paulus et al., 2006; Zeisberger et al., 2006; Zhang et al., 2010; DeNardo et al., 2011; Zhu et al., 2014). We chose clodronate liposomes for the depletion experiment due to the varied effects of anti-CSF1/CSF1R inhibition, including regulatory T cell (Treg) activation, recruitment of other myeloid populations, and resistance in certain macrophage subsets (Gyori et al., 2018; Kumar et al., 2017; Quail and Joyce, 2017; Zhang et al., 2020). Also, CSF1R is not specific to macrophages as it is a common marker for macrophage/dendritic cell (DC) progenitors, and the anti-CSF1R antibody has been shown to affect the DC population as well (Lohela et al., 2014). The effectiveness of the anti-CSF1R antibody varies among monocyte/macrophage (Mon/Mac) populations and proves ineffective against specific monocyte types (MacDonald et al., 2010; Zhang et al., 2020). Additionally, CSF1R blockade has been demonstrated to induce proinflammatory TAM phenotypes (Ao et al., 2017; Pyonteck et al., 2013). This could potentially lead to an undesirable proinflammatory environment within the tumor, which may not align with the objectives of our study.

**Figure 1.**
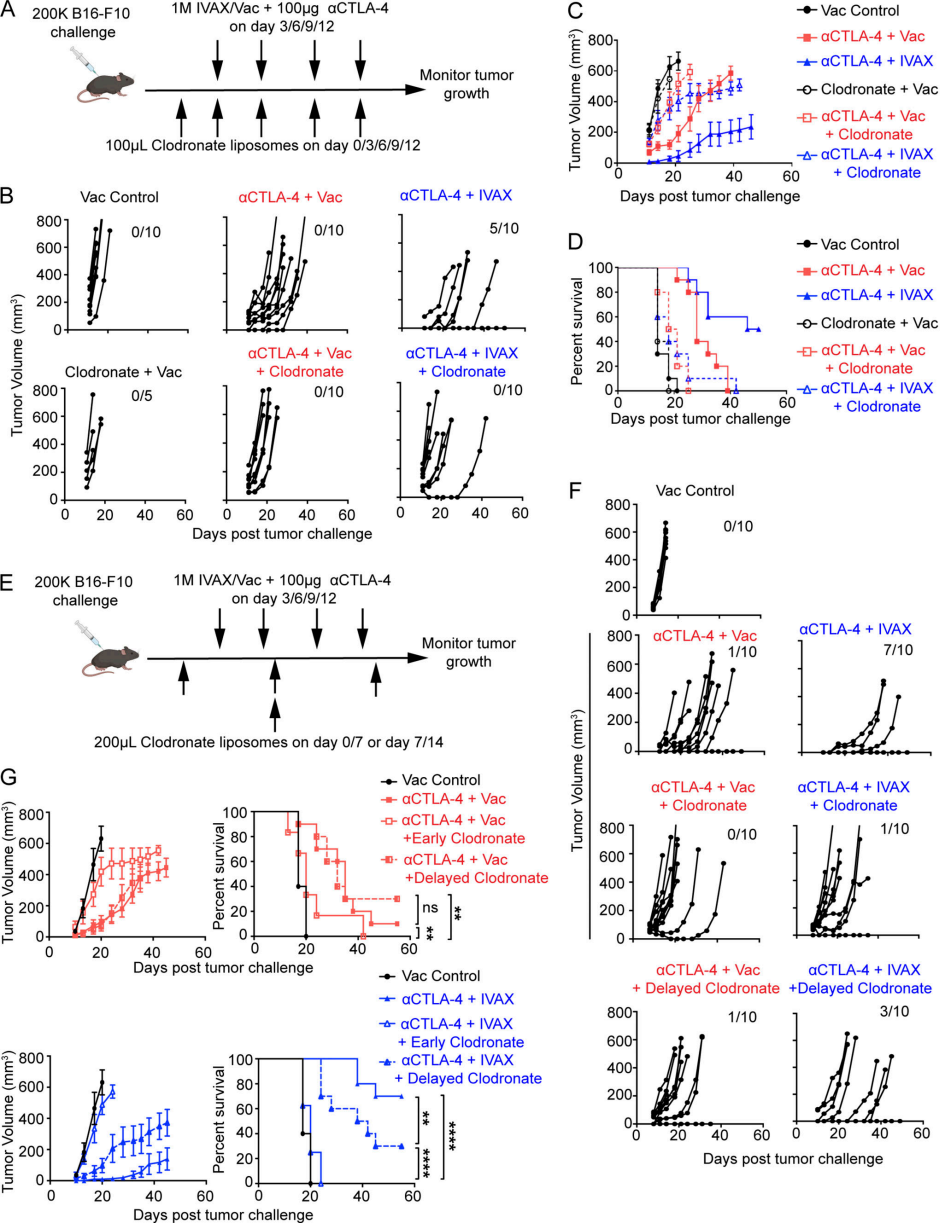
**Depletion of macrophages with clodronate liposomes abrogates tumor protection. (A)** Initial experiment setup with the treatment schedule. **(B)** Individual tumor growth curves after B16-F10 cells challenge. The numbers on the upper right side represent tumor-free mice. Data are representative of three independent experiments (*n* = 5–10 mice per group). **(C)** Tumor growth curves depict the average tumor volume in each group. Error bars represent means ± SEM. Data are representative of three independent experiments (*n* = 5–10 mice per group). **(D)** Survival curves represent three independent experiments (*n* = 5–10 mice per group). **(E)** Modified experiment setup with early vs. delayed treatment schedules. **(F)** Individual tumor growth curves after B16-F10 cells challenge. The numbers on the upper right side represent tumor-free mice. Data are representative of three independent experiments (*n* = 10 mice per group). **(G)** Cumulative average tumor growth curves (left panel) and survival curve (right panel) from two independent experiments (*n* = 10 mice per group). Error bars represent means ± SEM. Survival curves were analyzed with the log-rank test. ns, not significant; **P < 0.01; ****P < 0.0001. Irradiated parental B16 tumor cells (Vac) were used as a control for the IVAX group.

**Figure S2.**
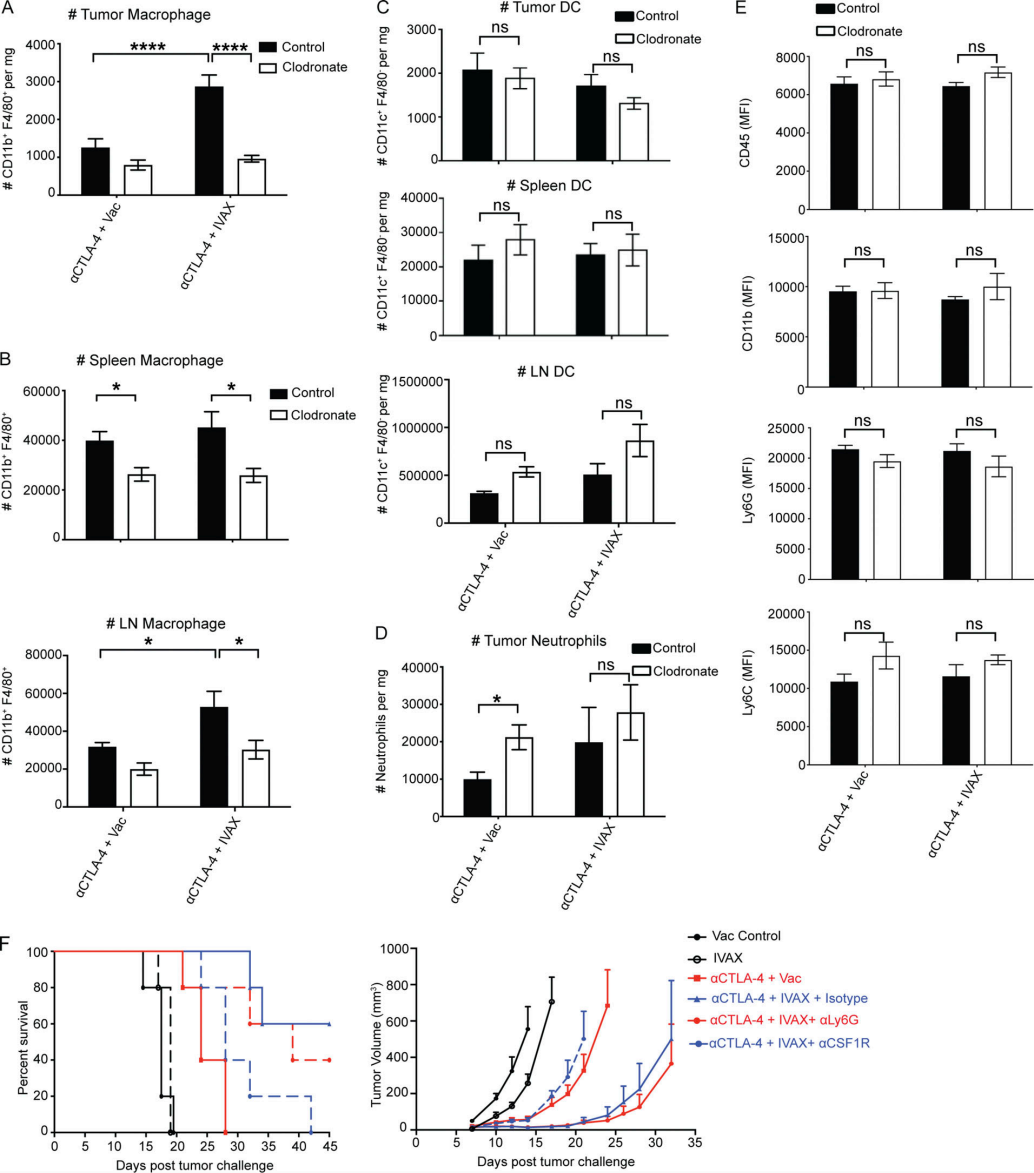
**Reduced intratumoral macrophages correlate with loss of tumor protection. (A)** The density of CD11b^+^ F4/80^+^ TAMs is depicted as an absolute number of cells per mg of tumor on day 16 after tumor challenge. Data were pooled from three independent experiments (*n* = 3 mice per group; one-way ANOVA, post hoc, ****P < 0.0001). **(B)** The density of CD11b^+^ F4/80^+^ TAMs is depicted as an absolute number of cells per mg of the spleen (upper panel) and a total number of CD11b^+^ F4/80^+^ TAMs in tumor-draining lymph nodes (lower panel) on day 16 after tumor challenge. Data were pooled from three independent experiments (*n* = 3 mice per group; one-way ANOVA, post hoc, *P < 0.05). **(C)** The density of CD11c^+^ F4/80^−^ DCs was depicted as an absolute number of cells per mg of tumor and spleen and a total number of CD11c^+^ F4/80^−^ DCs in tumor-draining lymph nodes on day 16 after tumor challenge. Data were pooled from three independent experiments (*n* = 3 mice per group; one-way ANOVA, post hoc, ns, not significant). **(D and E)** The density of neutrophils is depicted as an absolute number of cells per mg of tumor on day 16 after tumor challenge (D) and (E) the MFI of various surface receptors on the surface of neutrophils. Data were pooled from three independent experiments (*n* = 3 mice per group; one-way ANOVA, post hoc, ns, not significant *P < 0.05). **(F)** Mice were i.d. challenged on the right flank with B16-F10 tumor cells. Subsequently, i.p. injections of 100 µg anti-CTLA-4, combined with intradermal vaccination on the left flank with irradiated 10^6^ IVAX or irradiated parental B16 tumor cells, were administered on days 3, 6, 9, and 12. For specific cell depletion groups, as indicated, 500 µg of anti-Ly6G, anti-CSF1R, or isotype control antibodies were coadministered with the combination of IVAX plus anti-CTLA-4 on days 3, 6, 9, and 12. The data represent three independent experiments (*n* = 5 mice per group). Error bars indicate means ± SEM. Irradiated parental B16 tumor cells (Vac) were used as a control for the IVAX group.

We used clodronate liposomes to deplete macrophages and evaluated their impact on tumor growth. Mice received intraperitoneal (i.p.) injections of 0.5 mg clodronate every 3 days, starting from the day of tumor injection (day 0), in addition to CTLA-4 blockade monotherapy or combination therapy of IVAX and CTLA-4 blockade (Fig. 1 A). Macrophage depletion completely abrogated the enhanced protective benefits and the delay in tumor growth provided by IVAX to anti-CTLA-4 therapy (Fig. 1 B). The tumor grew much faster with macrophage depletion leading to all mice succumbing to tumor burden (Fig. 1, C and D). Recognizing the potential depletion of DCs by clodronate and the consequent implication on tumor antigen presentation, we delayed clodronate treatment to day 7 and limited the dosing to two injections of 1 mg on days 7 and 14 (Fig. 1 E). Given that clodronate liposomes delivered through the i.p. route need 2–3 days to take effect (Biewenga et al., 1995), we reason that this administration regimen would leave about a 10-day window for tumor antigen presentation and T cell priming and thus should minimize any effect on DCs. Early and delayed depletion both significantly impacted tumor progression. However, delayed depletion has a lesser effect, indicating clodronate’s effect on DCs can explain only part of the loss of therapeutic efficacy (Fig. 1 F). Early depletion resulted in similar tumor growth kinetics across groups with diminished therapeutic benefits. Interestingly, the delayed depletion selectively reduced tumor protection from IVAX and CTLA-4 blockade but not CTLA-4 alone (Fig. 1 G). The survival curves mirrored these trends, with delayed depletion minimally affecting anti-CTLA-4 monotherapy but significantly reducing the combination therapy efficacy (Fig. 1 G). While recognizing that the anti-CSF1R antibody may not represent the optimal approach for macrophage depletion, using the anti-CSF1R antibody as a proof of principle, we successfully depleted macrophages, observing similar impacts on IVAX and CTLA-4 blockade efficacy compared with clodronate liposomes (Fig. S2 F). Thus, macrophages play a critical role in the antitumor immunity generated by the combination of IVAX and CTLA-4 blockade.

### Reduced number of M1-like TAMs correlates with diminished tumor protection

We then isolated the tumor infiltrates and analyzed cell composition with flow cytometry. Tumors treated with IVAX and CTLA-4 blockade combination therapy were enriched in macrophages (Fig. S2 A). These increases were not observed in mice receiving clodronate liposomes under the aforementioned delayed treatment schedule, i.e., the density of macrophages in the tumor was significantly reduced (Fig. S2 A). Delayed treatment with clodronate liposomes caused a lesser degree of macrophage depletion in the spleen and tumor-draining lymph nodes (Fig. S2 B), unlike in the tumor, where clodronate induced a much more remarkable depletion of macrophages in the combination group (Fig. S2 A). Notably, delayed clodronate treatment had no significant impact on DCs in tumor, spleen, or tumor-draining lymph nodes (Fig. S2 C). This aligns with our conclusion that the diminished antitumor immunity after delayed clodronate treatment is predominantly due to macrophage depletion in tumors rather than the effects on the DC compartment. However, a recent study showed the impact of the clodronate liposomes on neutrophils, which are functionally arrested by this treatment (Culemann et al., 2023). Our experimental findings show that the administration of clodronate liposomes does not induce the depletion of neutrophils within the tumor model utilized in our study (Fig. S2 D). The study mentioned above also showed the change in the expression of surface markers, such as CD45, CD11b, Ly6C, and Ly6G, indicating the stunning of the neutrophils. Therefore, we also analyzed the expression of these markers and did not find a change in the expression of these receptors on neutrophils in clodronate-injected groups compared with the control, suggesting no such mechanism in our model (Fig. S2 E). The difference between our studies and this study could be due to the differences in the study models. Further, supporting the negligible role of neutrophils, depletion using an anti-Ly6G antibody did not affect the effectiveness of the IVAX and CTLA-4 blockade combination therapy (Fig. S2 F).

**Figure S3.**
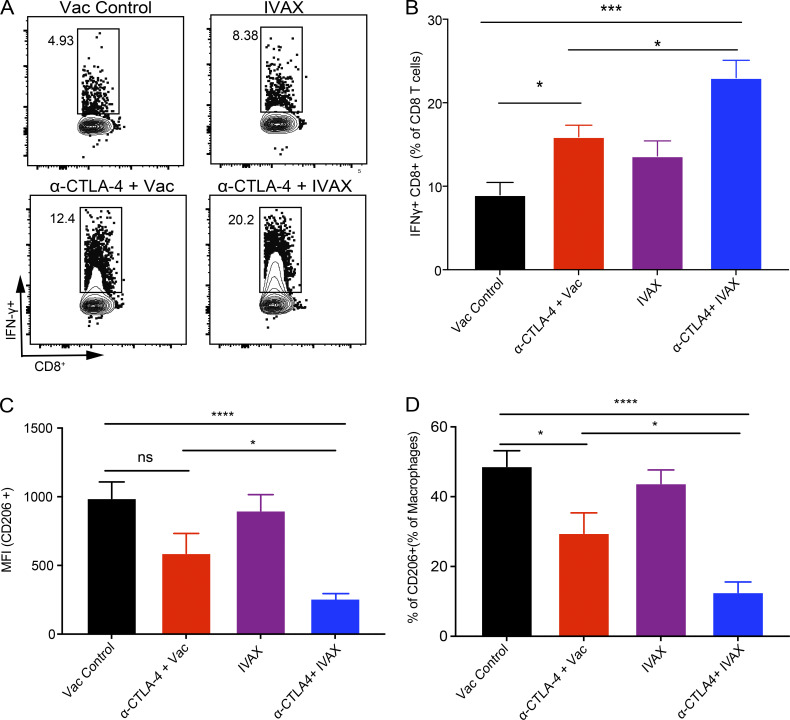
**Combination therapy reduces the suppressive efficacies of TAMs.** TAMs were isolated from tumors of indicated treatment groups and control as described in the Materials and methods. These macrophages were then incubated with naïve untouched total T cells isolated from the spleen with simultaneous in vitro stimulation with anti-CD3 (1.25 µg/ml) and anti-CD28 (1.25 µg/ml) antibodies for 48 h. Cells were stained with indicated antibodies. **(A and B)** Representative flow cytometry plots (A) and bar graphs (B) of IFN-γ in CD8 T cells. **(C)** MFI of CD206 expression on macrophages isolated from tumors of indicated treatment groups. **(D)** Frequencies of CD206^+^ macrophages in TAMs isolated from tumors of indicated treatment groups. Data represent two independent experiments (*n* = 5 mice per group; one-way ANOVA, post hoc, ns, not significant *P < 0.05; ***P < 0.001, ****P < 0.0001). Error bars indicate means ± SEM. Irradiated parental B16 tumor cells (Vac) were used as a control for the IVAX group.

We then analyzed the efficacy of TAMs from different treatment groups in suppressing T cell function. CD11b^+^ F4/80^+^ TAMs from all treatment groups were isolated by flow sorting and then incubated in vitro with naïve splenic conventional T cells, along with activation with anti-CD3 and anti-CD28 antibodies. Intracellular IFN-γ levels were analyzed after 48 h using flow cytometry. The results show that TAMs from the combination treatment group were less immunosuppressive, as indicated by higher IFN-γ secretion from CD8 T cells in their presence than the TAMs from other treatment groups (Fig. S3, A and B). Additionally, a decrease in the mean fluorescence intensity (MFI) of CD206 on TAMs and a decrease in frequencies of CD206^+^ TAMs in the combination treatment group suggested that TAMs are skewed to a less immunosuppressive phenotype (Fig. S3, C and D).

### Tumor-infiltrating immune cells identified by scRNA-seq

**Figure S4.**
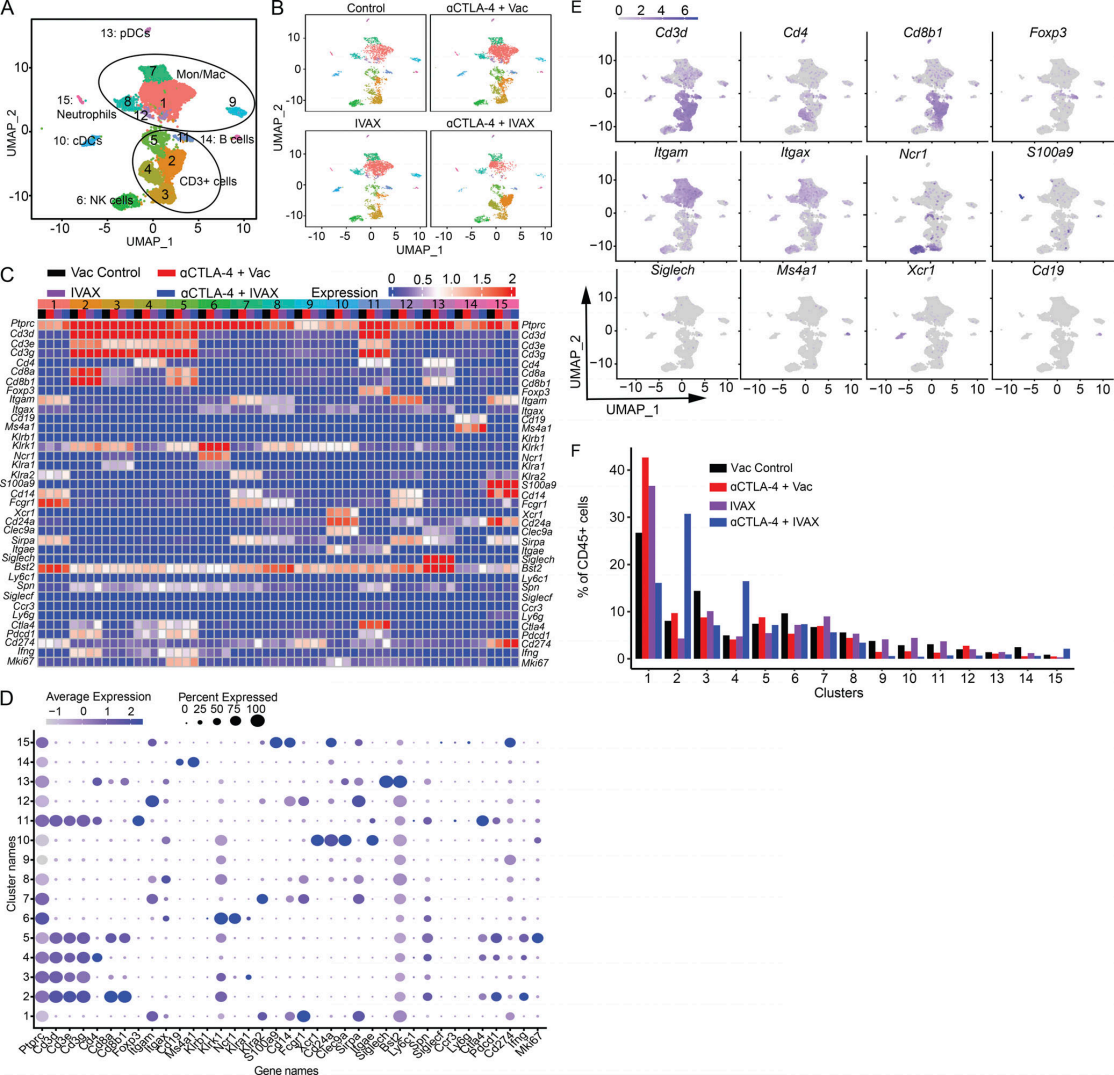
**scRNA-seq analysis of changes in immune cell composition in tumors of different treatment groups.** Mice were challenged with B16-F10 tumors and were given indicated treatments; tumors were isolated and digested. TILs from tumors were isolated and stained with anti-CD45.2 antibody for sorting by FACS, and a 10X library was prepared and analyzed as described in the Materials and methods. **(A)** UMAP graph showing the clusters and annotation. **(B)** UMAP graph showing the clusters in each treatment group. **(C)** Heatmap displays selected marker expressions for each cluster. **(D)** Dot plots show the differential gene expression of cell-specific lineage markers in different clusters. **(E)** UMAP graphs showing the expression of selected markers. **(F)** Bar plot of the frequency of each cluster. Cluster names are indicated on the x axis, and frequencies of each cluster are on the y axis. Data are representative of two independent experiments (*n* = 5 mice per group). Mice within each group were polled for analysis. Irradiated parental B16 tumor cells (Vac) were used as a control for the IVAX group.

ICOS is a member of the CD28 family and is one of the costimulatory molecules upregulated upon T cell activation. The ICOS-mediated signal is involved in regulating activated T cells and effector T cell (Teff) functions through PI3K signaling. To evaluate the role of ICOS signaling in the combination of IVAX and CTLA-4 blockade on the intratumoral cell composition and immune cell differentiation, we employed scRNA-seq and mass cytometry (CyTOF) for high-dimensional profiling. Initially, we analyzed changes in the tumor-infiltrating immune cell population at the single-cell level by scRNA-seq. To that end, mice were injected with B16-F10 cells intradermally (i.d.) and later treated with a combination of anti-CTLA-4 and vaccine comprising irradiated ICOSL-negative B16-F10 cells (Vac) or ICOSL-positive B16-F10 cells (IVAX). On day 16 of the post-tumor challenge, tumors were isolated and digested, and CD45^+^ cells were sorted using FACS. The sequencing aimed to capture 8,000–10,000 cells per sample, with a target coverage of 30,000–50,000 mean reads per cell. 15 *ptprc*-positive (CD45^+^) clusters were identified using a clustering algorithm that optimized modularity based on shared nearest neighbors and the first 50 principal components as described in methods (Fig. S4, A–D). Analysis of these clusters using the ImmGen database (Aran et al., 2019; Heng et al., 2008) and by known cell-type markers revealed five clusters of Mon/Mac, five T cell clusters, one classical DC (cDC) cluster, one plasmacytoid DC (pDC) cluster, one NK cell cluster, one neutrophil cluster, and one B cell cluster (Fig. S4, A and C–E). We found that the combination therapy modulates many tumor-infiltrating cell populations, especially T cell and macrophage populations, compared with anti-CTLA-4 treatment alone (Fig. S4, B and F).

### Analysis of remodeling of T cell compartments by scRNA-seq

**Figure 2.**
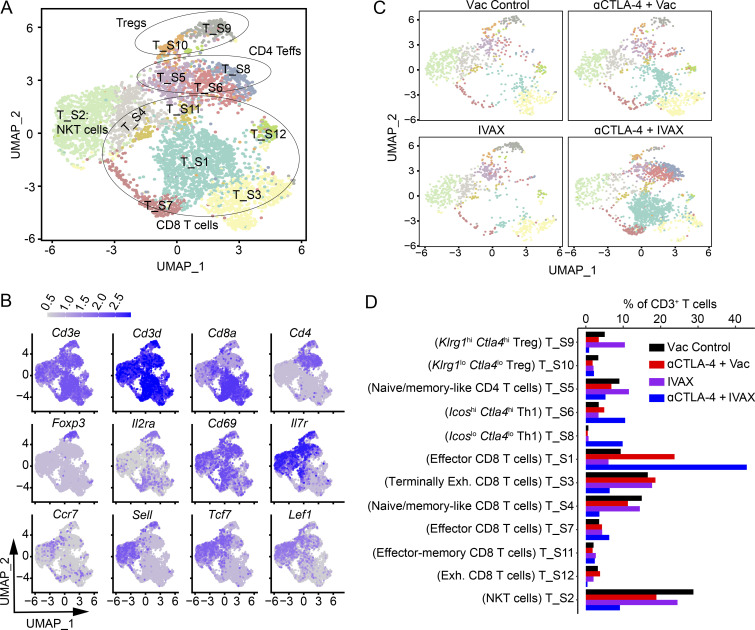
**scRNA-seq analysis of T cell subsets and their heterogeneity in tumors of different treatment groups.** Mice were challenged with B16-F10 cells and were given indicated treatments; tumors were isolated and digested. TILs from tumors were isolated and stained with anti-CD45.2 antibody for sorting by FACS, and a 10X library was prepared and analyzed as described in the Materials and methods. The CD3^+^ cells were reclustered, and T cell subpopulations were characterized. **(A)** UMAP graph showing the clusters and annotation. **(B)** UMAP graphs showing the expression of selected markers. **(C)** UMAP graph showing the clusters in each treatment group. **(D)** Bar plot of the frequency of each T cell cluster. Cluster names are indicated on the y axis and frequencies on the x axis. Data represent two independent experiments (*n* = 5 mice per group). Mice within each group were pooled for analysis. Irradiated parental B16 tumor cells (Vac) were used as a control for the IVAX group.

ICOS, expressed by various T cell subsets, plays an important role in the efficacy of anti-CTLA-4 and IVAX combination therapy (Fan et al., 2014). Observing changes in T cell clusters (Fig. S4, B and F), we reclustered CD3^+^ (*Cd3e*^+^ and *Cd3d*^+^) clusters (cluster numbers 2–5 and 11) and analyzed changes in T cell subsets among treatment groups at higher resolution. The clusters were annotated based on expression levels of classical T cell subset markers *Cd3e*, *Cd3d*, *Cd8a*, *Cd4*, *Foxp3*, *Il2ra*, *Cd69*, and *Klrb1c*, etc., and the level of expression of various functional and costimulatory molecules *Gzmb*, *Ifng*, *Prf1*, *Icos*, *Pdcd1*, *Lag3*, etc. (Fig. 2, A–D). We revealed 12 clusters of CD3 T cells, which included two clusters of Tregs (T_S9 and T_S10), three clusters of CD4 effector T cells (CD4 Teffs) (clusters T_S5, T_S6, and T_S8), six clusters of CD8 T cells (T_S1, T_S3, T_S4, T_S7, T_S11, and T_S12), and one natural killer T (NKT) cell cluster (cluster T_S2) (Fig. 2, A–D). Treg cluster T_S9 differs from cluster T_S10 in high expression of *Klrg1*, *Ctla4*, *Foxp3*, *Il2ra*, *Icos*, etc. (Fig. 2, A and D). We found that of the two Treg subsets, cluster T_S9 showed a decrease in frequency in the combination treatment group compared with the anti-CTLA-4 treatment alone. Amongst three clusters of CD4 Teff cells, T_S6 and T_S8 increased in frequency in the combination treatment group compared with the other groups (Fig. 2, C and D). Clusters T_S6 and T_S8 were positive for *Cxcr3*, *Ifng*, *Icos*, and *Cd69* and therefore annotated as Th1 CD4 T cells. Cluster T_S8 was distinguishable from T_S6 by the higher expression of *Tnfrsf4*, *Icos*, *Ifng*, and *Ctla4*. Cluster T_S5 decreased in frequency in the combination treatment group compared with the anti-CTLA-4 treatment group. This cluster was annotated as a naïve/memory-like CD4 T cell cluster as it expressed *Tcf7* and had a high expression of *Il7r* but a low *Icos* expression. Among CD8 T cell clusters, T_S3, T_S4, and T_S12 decreased in frequency in the combination treatment group compared with the other groups (Fig. 2, C and D). CD8 T cell cluster T_S3 was positive for *Ctla4*, *Lag3*, and *Havcr2*, and expressed a high level of *Pdcd1*, *Tox*, and *Gzb*, but no expression of *Icos*, and represented terminally exhausted CD8 T cells. Cluster T_S4 had high expression of *Tcf7* and *Il7r* and was positive for *Lef1* and *Sell*. This cluster was annotated as a naïve/memory-like cluster. Cluster T_S12 was positive for *Ctla4* and *Havcr2* and expressed high *Pdcd1*, *Gzmb*, and *Mki67* but low *Tox*. This cluster was annotated as exhausted CD8 T cells. The CD8 T cell clusters that increased after the combination treatment included clusters T_S1 and T_S7, and both these clusters were annotated as effector CD8 T cells (Fig. 2, A and D). Cluster T_S1 was positive for *Cxcr3*, *Cd69*, and *Ifng* and expressed high levels of *Cd28*, *Cd27*, *Icos*, *Lag3*, *Pdcd1*, and *GzB*, whereas cluster T_S7 displayed low *Ctla4*, *Pdcd1*, *Lag3*, and *Tox*, but high expression of *Cd28*, *Cd27*, *Tnfrsf9*, *Xcl1*, *GzB*, and *Ifng*. Cluster T_S11 did not change after the combination therapy treatment and was identified as an effector-memory cluster with positive expression of *Lef1* and *Sell* and high expression of *Tcf7*, *Il7r*, *Cd69*, and *Gzmb*. Therefore, our data show that among CD4 T cells, there was an increase in Th1 effector CD4 T cells in the combination therapy group compared with the anti-CTLA-4 treatment group alone. Among CD8 T cell subsets, the data show an increase in effector CD8 T cell clusters in the combination therapy group. On the other hand, we found a decrease in clusters annotated as terminally exhausted, exhausted, and naïve/memory-like CD8 T cells. Thus, an increase in CD4 Th1 cells and effector CD8 T cells and a decrease in Treg cells and exhausted CD8 T cells mark modulation of the T cell compartment toward antitumor phenotype.

**Figure S5.**
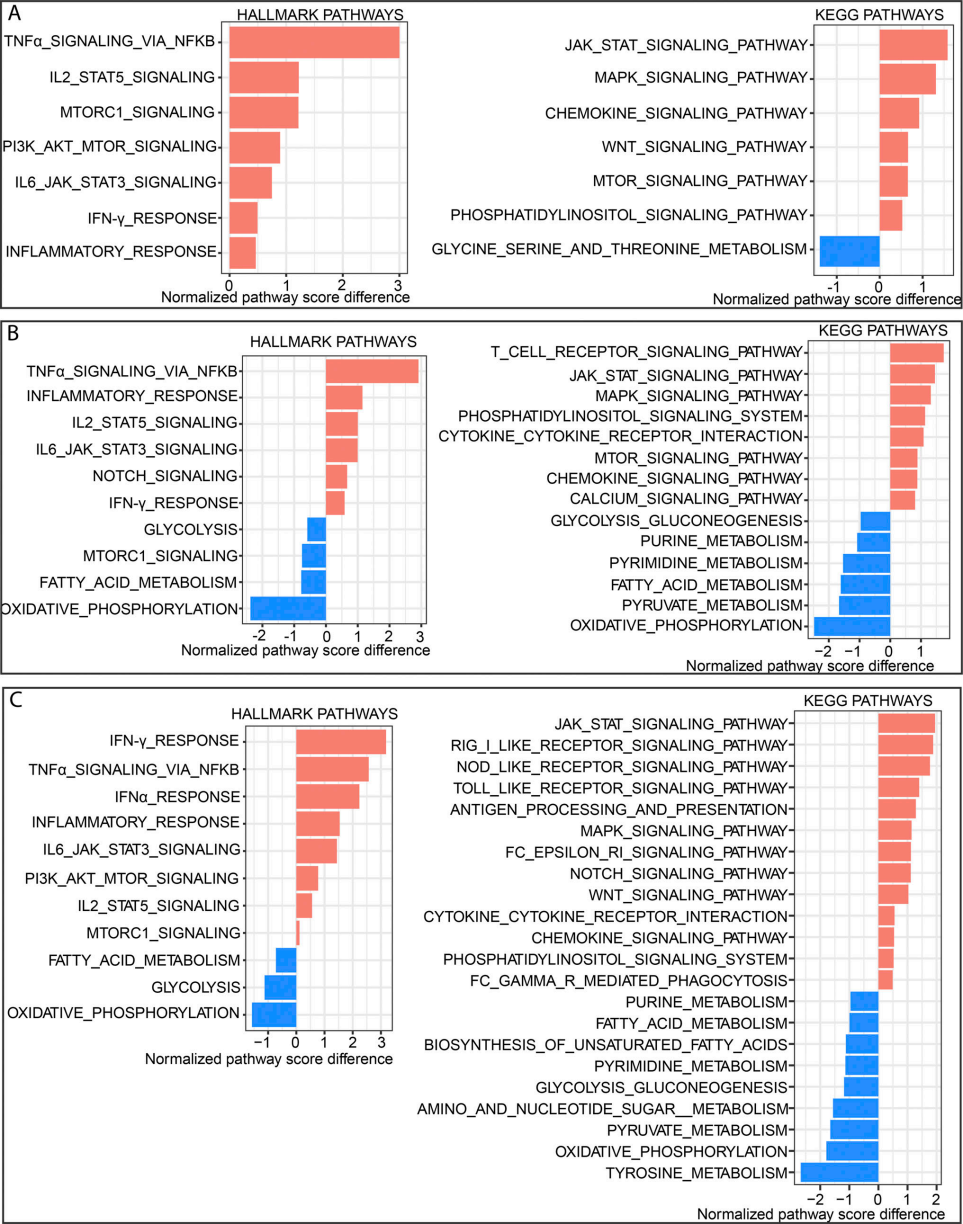
**Single-cell analysis of metabolic pathway activity of tumor-infiltrating immune cells in combination therapy compared with anti-CTLA-4 antibody.** The bar graph shows normalized pathway enrichment score differences for each particular cell type, which are calculated by subtracting the median pathway enrichment score in the anti-CTLA-4 from that of the combination therapy. **(A–C)** Hallmark and KEGG analysis of differential pathway activities in a combination therapy group (A) CD4 Teff cells, (B) CD8 T cells, and (C) macrophages.

It has been previously reported that ICOS activation increases calcium mobilization induced by the T cell receptor and activates PI3K signaling in T cells (Chen et al., 2014; Fos et al., 2008; Leavenworth et al., 2015; O’Brien and Harris, 2020); therefore, we wanted to analyze the pathways activated by ICOS signaling. To that end, we used KEGG and Hallmark pathways analysis to reveal the enrichment of immune-related or inflammatory pathways in the combination group compared with anti-CTLA-4 alone in T cells and TAMs (Fig. S5). Among CD4 Teff and CD8 T cells, there was a significant upregulation of TNF-α signaling, IL2 signaling, IL6 signaling, IFN-γ response, chemokine signaling, Jak-stat signaling, MAPK signaling, mTOR, inflammatory response pathways, calcium signaling, chemokine signaling, and PI3K signaling pathways in combination therapy (Fig. S5, A and B). Additionally, T cell receptor pathways increased specifically in CD8 T cells (as observed in CD8 T cells; Fig. S5 B). These pathways are associated with T cell activation and antitumor immunity. On the other hand, there was a decrease in glycine, serine, threonine (in CD4 Teffs), glycolysis, fatty acid, purine, pyrimidine, pyruvate, and oxidative phosphorylation (OXPHOS) pathways in the combination treatment group compared with anti-CTLA-4 alone. The metabolic activity of T cell subsets is unique and dependent upon the differentiation state. There is a slight decrease in some of these metabolic pathways, but OXPHOS is most decreased in CD8 T cells in the combination treatment compared with anti-CTLA-4 alone treatment. In melanoma patients, the presence of a CD8 T cell subset that exhibits high OXPHOS is indicative of resistance to immunotherapy (Li et al., 2022). This suggests that there is a decrease in resistance pathways in combination therapy. The reduction in other metabolic pathways, such as glycolysis, is not considerable, but it’s surprising considering that the T cells exhibit more effector phenotype in combination therapy. These cells may be in the transitioning mode for the reversal of these pathways.

### CyTOF analysis of the T cell compartment in the combination of ICOS engagement and CTLA-4 blockade

**Figure 3.**
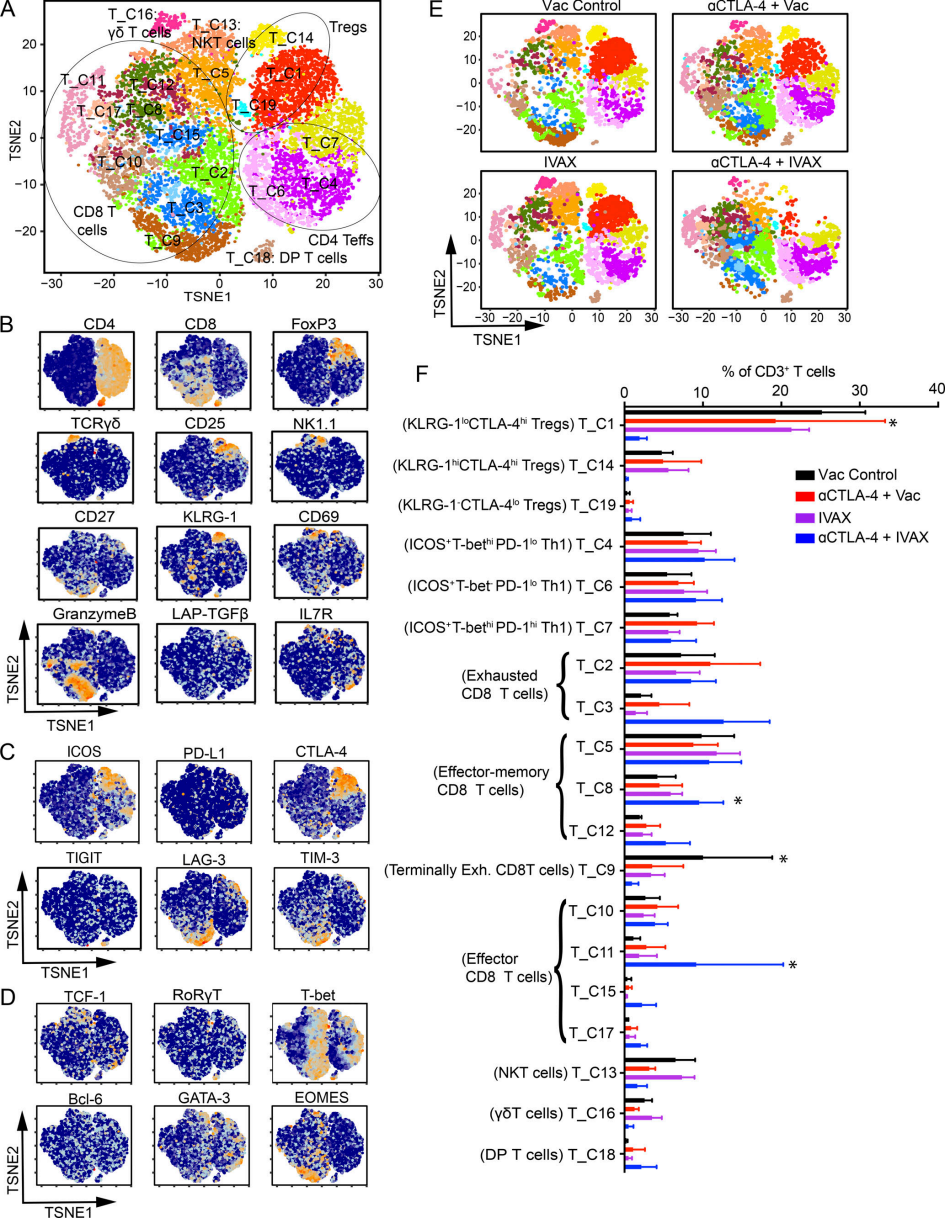
**Characterization of intratumoral T cell subtypes and their heterogeneity by CyTOF.** CyTOF proteomic analysis using a T cell antibody panel for TILs from mice challenged with B16-F10 cells and given indicated treatments as described in the Materials and methods. **(A)** t-SNE plot of an equal number of tumor-infiltrating CD3 T cells from each group and overlaid with color-coded clusters. **(B–D)** Expression of (B) T cell signature genes, (C) T cell cosignaling receptors, and (D) transcription factors projected onto t-SNE plot in A. **(E)** t-SNE plots with clusters of T cells in each indicated treatment group. **(F)** Bar plot of the frequency of each T cell cluster. Cluster names are indicated on the y axis, and frequencies of each cluster are on the x axis. Data represent three independent experiments (*n* = 4–7 mice per group; *t* test, *P < 0.05), and significance is shown between αCTLA-4 and αCTLA-4 + IVAX treatment groups. Error bars represent means ± SEM. Irradiated parental B16 tumor cells (Vac) were used as a control for the IVAX group.

We then investigated the effects of combination therapy on T cell modulation by mass cytometry (CyTOF) to assess markers’ protein expression. 19 T cell clusters were detected within tumor-infiltrating CD3 T cells at a relative frequency of over 0.5% (Fig. 3, A and F). They were annotated as three clusters of Tregs (clusters T_C1, T_C14, and T_C19), three clusters of CD4 Teff cells (clusters T_C4, T_C6, and T_C7), 10 clusters of CD8 T cells (T_C2, T_C3, T_C5, T_C8 to T_C12, T_C15, and T_C17), one cluster of NKT cells (cluster T_C13), one γδ T cell cluster (cluster T_C16), and one double-positive T cell cluster (DP T cells) expressing CD4 and CD8 (cluster T_C18). Among Treg clusters, cluster T_C1 was FoxP3^hi^ KLRG-1^lo^ CTLA-4^hi^ LAP-TGFβ^hi^ and cluster T_C14 was FoxP3^hi^ KLRG-1^hi^ CTLA-4^hi^ LAP-TGFβ^hi^. Cluster T_C1 and T_C14 differ in expression of KLRG-1, and these Treg clusters decreased in frequencies in the combination treatment group compared with other treatment groups, but only a decrease in cluster T_C1 was statistically significant (Fig. 3, E and F). The high expression of CTLA-4, Foxp3, and LAP-TGFβ suggests that these Treg clusters are more immunosuppressive (Fig. 3, B, C, and F). On the other hand, cluster T_C19, a FoxP3^lo^ KLRG-1^−^ CTLA-4^lo^ LAP-TGFβ^lo^ Treg cluster, showed a slight non-significant increase in the combination treatment group compared with anti-CTLA-4 monotherapy. Interestingly, there was a decrease in the immunosuppressive Treg clusters in the combination treatment group compared with the anti-CTLA-4 alone treatment group (Fig. 3, E and F). Among CD4 Foxp3^-^ Teff cell clusters, clusters T_C4 and T_C6 showed a non-significant slight increase in frequency in the combination treatment group (Fig. 3 F). Cluster T_C4 is CXCR3^+^ ICOS^+^ PD-1^lo^ TIM-3^−^ LAG-3^−^ T-bet^hi^ CD4 T cells and cluster T_C6 is CXCR3^+^ ICOS^+^ PD-1^lo^ TIM-3^−^ LAG-3^−^ T-bet^−^ CD4 T cells. These clusters were annotated as CD4 Th1 cells as they expressed CXCR3 and ICOS, and either they lacked inhibitory receptors or expressed them at low levels (Bonecchi et al., 1998; Sallusto et al., 1998). CD4 Th1 subset has been shown to play an antitumor role, and an increase in CD4 Th1 could contribute to the antitumor efficacy of the combination therapy (Borst et al., 2018). Among CD8 T cell clusters, clusters T_C2 and T_C3 were annotated as exhausted CD8 T cells. These clusters do not show statistically significant changes in the combination therapy group compared to the anti-CTLA-4 alone therapy group (Fig. 3 F). Clusters T_C5, T_C8, and T_C12 are annotated as effector-memory CD8 T cells. Cluster T_C5 expresses higher levels of IL7R, TCF-1, and CD44 than T_C8 and T_C12. T_C12 expresses the lowest of these receptors among these three clusters of effector-memory CD8 T cells. Of these, T_C5 did not change in the combination therapy compared to the anti-CTLA-4. On the other hand, there was an increase in clusters T_C8 and T_C12, but a significant increase only in cluster T_C8 (Fig. 3 F). Clusters T_C10, T_C11, T_C15, and T_C17 were annotated as effector CD8 T cells. Cluster T_C15 has the highest expression of KLRG-1, CD44, T-bet, and granzyme B compared with the other three effector CD8 T cell clusters. Cluster T_C10 has higher CD44, PD-1, and CD27 but lower T-bet expression than cluster T_C11. Clusters T_C11, T_C15, and T_C17 showed increased frequency, whereas cluster T_C10 remained unchanged in the combination therapy group. However, only the increase in the frequency of cluster T_C11 was statistically significant. The cluster T_C9 was annotated as PD-1^hi^ LAG-3^hi^ TIM-3^hi^ EOMES^hi^ terminally exhausted CD8 T cells, and this cluster showed a statistically significant decrease in frequency in the combination therapy group. These results indicate that combination treatment decreases Treg frequency and modulates CD4 Teff cells toward a more Th1 effector phenotype. Among CD8 T cells, there is a significant increase in effector CD8 T cells, whereas terminally exhausted CD8 T cells decreased in frequency in the combination therapy group compared with anti-CTLA-4 therapy alone.

### scRNA-seq reveals remodeling of tumor-infiltrating myeloid cells in combination therapy

**Figure 4.**
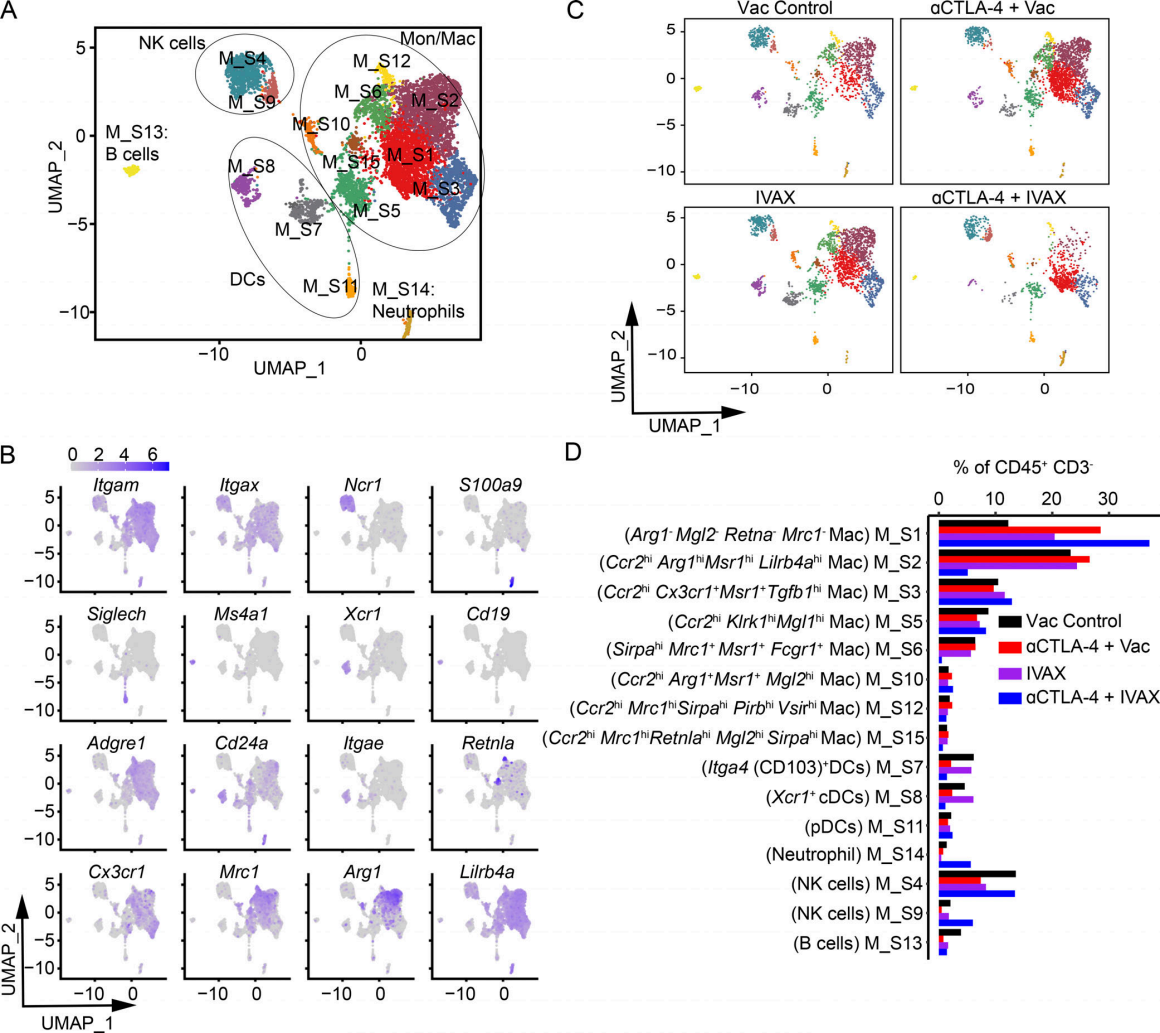
**scRNA-seq analysis of CD45**^**+**^ **CD3**^**−**^ **cell subsets and their heterogeneity in tumors of different treatment groups.** Mice were challenged with B16-F10 cells and were given indicated treatments; tumors were isolated and digested. TILs from tumors were isolated and stained with anti-CD45.2 antibody for sorting by FACS, and a 10X library was prepared and analyzed as described in the Materials and methods. The *Ptprc *(CD45)^+^ CD3^−^ cells were reclustered and intracellular myeloid subsets were characterized. **(A)** UMAP graph showing the clusters and annotation. **(B)** UMAP graphs showing the expression of selected markers. **(C)** UMAP graph showing the clusters in each treatment group. **(D)** Bar plot of the frequency of each cluster. Cluster names are indicated on the y axis, with frequencies on the x axis. Data represent two independent experiments (*n* = 5 mice per group). Mice within each group were pooled for analysis. Irradiated parental B16 tumor cells (Vac) were used as a control for the IVAX group.

Myeloid cells are one of the most abundant cell types in the TME, and we observed modulation of myeloid cells in our dataset (Fig. S4, B and F). Therefore, we investigated the heterogeneity of myeloid cells in tumors of different treatment groups by finer clustering. The *ptprc* (CD45)^+^ CD3^− ^clusters were reclustered, which produced 15 clusters that were annotated as eight Mon/Mac clusters (clusters M_S1, M_S2, M_S3, M_S5, M_S6, M_S10, M_S12, and M_S15), three DC clusters (clusters M_S7, M_S8, and M_S11), one neutrophil cluster (cluster M_S14), two NK cell clusters (clusters M_S4 and M_S9), and one B cell cluster (cluster M_S13) (Fig. 4, A and D). Among monocyte–macrophage clusters, clusters M_S2, M_S6, M_S12, and M_S15 decreased in the combination treatment group, whereas cluster M_S1 was the only cluster that increased in the combination therapy group compared to other groups (Fig. 4, C and D). To better understand the potential functions of diverse clusters of macrophages, we evaluated the expression of classical M1 and M2-type macrophage markers in these macrophage clusters. Among clusters that decreased in frequency in the combination treatment group, M_S2 expressed *Ccr2*, with relatively higher expressions of *Arg1*, *Msr1*, and *Lilrb4a* (Fig. 4 D). Cluster M_S6 expressed *Msr1*, *Mrc1*, and *Fcgr1* and had a relatively high expression of *Sirpa*. Cluster M_S12 expressed *Msr1* and relatively high levels of *Ccr2*, *Mrc1*, *Sirpa*, *Vsir*, and *Pirb*. Cluster M_S15 expressed high levels of *Ccr2*, *Mgl2*, *Retnla*, *Mrc1*, and *Sirpa*. These clusters express genes such as *Msr1*, *Mrc1*, *Sirpa*, *Arg1*, *Cx3cr1*, *Lilrb4a*, *Vsir*, and *Pirb*, which are associated with immunosuppressive macrophages (Biswas and Mantovani, 2010; Gubin et al., 2018; Murray et al., 2014; Qian and Pollard, 2010; Sharma et al., 2021). Cluster M_S1, which increased in the combination therapy group compared with other groups, did not express *Arg1*, *Mgl2*, *Retnla*, and *Mrc1* and expressed low levels of *Msr1*, *Pirb*, and *Lilrb4a*. This cluster lacks genes associated with immunosuppressive macrophages or expresses them at a low level, exhibiting an antitumor phenotype. Macrophages are plastic cells, and current studies have shown that they exist in a continuum rather than M1 and M2 binary states. Our data also suggest the continuum of macrophages with markers intersecting M1 and M2 phenotypes. However, our data show a decrease in the frequency of macrophage clusters that displayed markers associated with immunosuppressive phenotype in combination therapy and an increase in the cluster that did not display those markers.

Among macrophages, there was a significant upregulation of several signaling pathways and immune responses, including RIG-I-like receptor, NOD-like receptor, Toll-like receptor (TLR), antigen processing and presentation, TNF-α, IFN-γ response, IFN-α response, Jak-stat, MAPK, mTOR, inflammatory response, chemokine, FcγR-mediated phagocytosis, and PI3K in combination therapy (Fig. S5 C). Many of these pathways are antitumor immune pathways involved in T cell activation, Teff differentiation, and macrophage differentiation to proinflammatory or antitumor macrophages. Therefore, the data suggest increased antitumor pathways with combination therapy, confirming our scRNA-seq and ex vivo suppression data. Further, data show that most metabolic pathways, such as OXPHOS and glycolysis, are reduced in the macrophages in combination therapy. M1 macrophages have decreased OXPHOS and increased dependence on glycolysis (Kelly and O’Neill, 2015). So, the reduced OXPHOS does suggest skewing toward M1-type macrophages. Also, the decreased glycolysis could be the result of a transitory phenotype. However, inhibition of glycolysis has been shown to reduce the expression of Arg1 and CD206, the hallmarks of M2 macrophages, decreasing the M2 macrophage polarization (Zhao et al., 2017). We also see a decrease in fatty acid metabolism, and evidence has shown that fatty acid metabolism may contribute to M2 activation by fueling OXPHOS. The protumor functions and generation of TAMs can be abolished by inhibiting lipid uptake or fatty acid oxidation in macrophages (Su et al., 2020). Therefore, a decrease in fatty acid metabolism in combination therapy shows more of an M1 phenotype.

### CyTOF analysis reveals remodeling of the myeloid compartment in combination therapy

**Figure 5.**
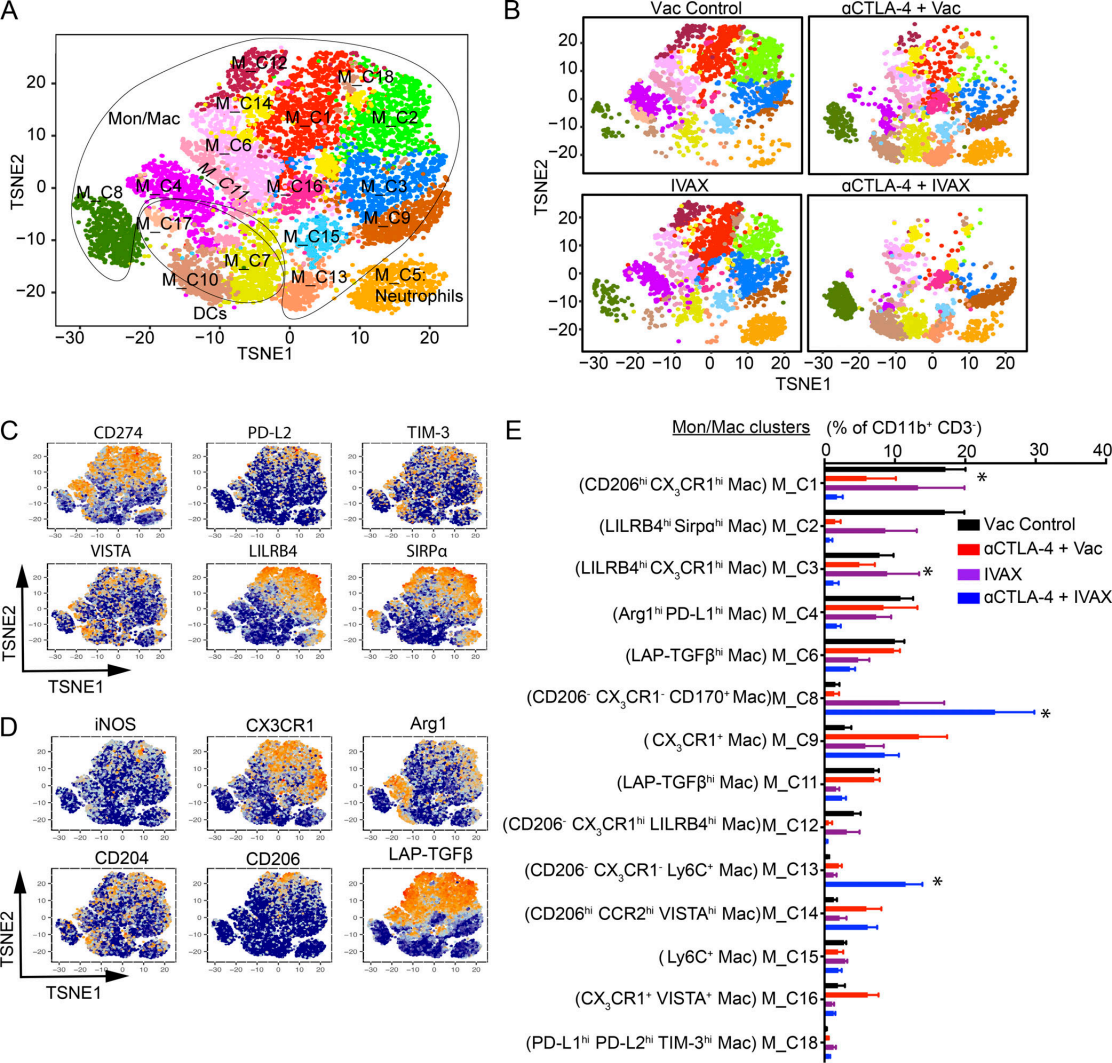
**Combination therapy decreased the frequency of myeloid clusters associated with immunosuppressive phenotypes.** CyTOF proteomic analysis using a myeloid cell antibody panel for TILs from mice challenged with B16-F10 cells and given indicated treatments as described in the Materials and methods. **(A)** t-SNE plot of an equal number of CD11b^+^ CD3^−^ TILs from each group and overlaid with color-coded clusters. **(B–D)** t-SNE plots with clusters of intratumoral CD11b^+^ CD3^−^ cells in each treatment group. Expression of (C) inhibitory receptors and (D) M1/M2 macrophage markers projected onto t-SNE plot in A. **(E)** Bar plot of the frequency of each cluster. Cluster names are indicated on the y axis, and frequencies of each cluster are on the x axis. Data represent three independent experiments (*n* = 4–7 mice per group; *t* test, *P < 0.05), and significance is shown between αCTLA-4 and αCTLA-4 + IVAX treatment groups. Error bars represent means ± SEM. Irradiated parental B16 tumor cells (Vac) were used as a control for the IVAX group.

After analyzing the tumor-infiltrating myeloid population changes by single-cell RNA expression analysis, we explored the macrophage population changes by CyTOF, which probes the protein expression on the surface. To this end, mice were treated with combination therapy on different days and tumors were dissected; cells were isolated from tumors, stained with CyTOF antibodies, and analyzed by high-parameter CyTOF (Helios). 18 clusters were identified amongst CD11b^+^ CD3^−^ tumor-associated cells with relative frequencies >0.5% (Fig. 5 A). These clusters were annotated as 14 clusters of Mon/Mac (clusters M_C1 to M_C4, M_C6, M_C8, M_C9, M_C11 to M_C16, and M_C18), three DC clusters (M_C7, M_C10, and M_C17), and one neutrophil cluster (cluster M_C5) (Fig. 5, A and E). Amongst Mon/Mac clusters, M_C1 to M_C4, M_C6, and M_C11 showed a decrease in frequency in combination treatment compared to the anti-CTLA-4 treatment control (Fig. 5, B and E). The expression of CD206, CD204, LAP-TGFβ, and CX_3_CR1 in these clusters suggests that these clusters are suppressive macrophage clusters (Fig. 5, C–E). However, out of these clusters, only M_C1 and M_C3 had a significant decrease in frequencies after the treatment with combination therapy compared with anti-CTLA-4 treatment control (Fig. 5 E). Cluster M_C1 was CD11b^+^ F4/80^hi^ CD68^hi^ CD206^hi^ CX_3_CR1^hi^ CD204^+^ VISTA^+^ LILRB4^+^ PD-L1^+^ LAP-TGFβ^+^ cluster, and cluster M_C3 was CD11b^+^ F4/80^+^ CD68^hi^ CX_3_CR1^hi^ LILRB4^hi^ PD-L1^+^ LAP-TGFβ^+^ cluster (Fig. 5 E). The macrophage clusters M_C8 and M_C13 increased after the combination therapy treatment. Cluster M_C8 is CD11b^+^ F4/80^+^ CD170^+^ CD68^−^ CD206^−^ LILRB4^−^ CX_3_CR1^−^ cluster whereas cluster M_C13 is CD11b^lo^ F4/80^lo^ CD68^−^ CD206^−^ LILRB4^-^ CX_3_CR1^−^ Ly6C^+^ cluster (Fig. 5 E). These clusters did not express phenotypes associated with suppressive macrophages and, therefore, interestingly, a reduction in the levels of suppressive phenotype macrophage clusters and an increase in non-suppressive macrophage clusters suggest the change in TME from protumor to antitumor after combination treatment.

These findings and scRNA-seq and ex vivo suppression data suggest that the large number of macrophage clusters that are reduced by the combination of IVAX and anti-CTLA-4 are immunosuppressive TAMs, and the clusters that increase in population after the treatment may be M1-like antitumor macrophages. These data also raise the possibility that the proinflammatory functions of these type 1 macrophages contribute to the tumor protection effect from combination therapy of CTLA-4 blockade and ICOS engagement.

### Macrophage depletion is accompanied by profound changes in T cells in the tumor

**Figure 6.**
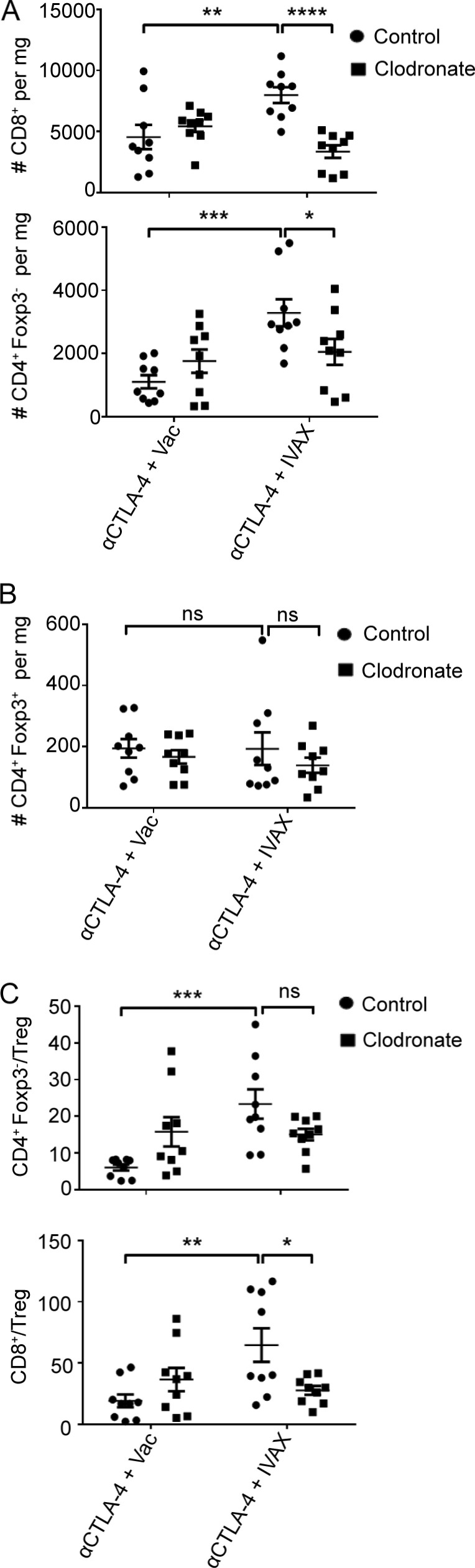
**Macrophage depletion is accompanied by a reduction of T cell infiltration. (A)** The densities of CD8 and CD4^+^ Foxp3^−^ effector T cells (CD4 Teffs) were depicted as an absolute number of cells per mg of tumor on day 16 after tumor challenge. Data were pooled from three independent experiments (*n* = 3 mice per group; one-way ANOVA, post hoc, *P < 0.05; **P < 0.01; ***P < 0.001, ****P < 0.0001). **(B)** The density of CD4^+^ Foxp3^+^ Tregs was depicted as an absolute number of cells per mg of tumor on day 16 after tumor challenge. Data were pooled from three independent experiments (*n* = 3 mice per group; one-way ANOVA, post hoc, ns, not significant). **(C)** Quantification of CD8/Treg and CD4^+^ Foxp3^−^ Teff/Treg ratios in day 16 B16-F10 tumors. Data were pooled from three independent experiments (*n* = 3 mice per group; one-way ANOVA, post hoc ns, not significant; *P < 0.05; **P < 0.01; ***P < 0.001). Error bars represent means ± SEM. Irradiated parental B16 tumor cells (Vac) were used as a control for the IVAX group.

**Figure 7.**
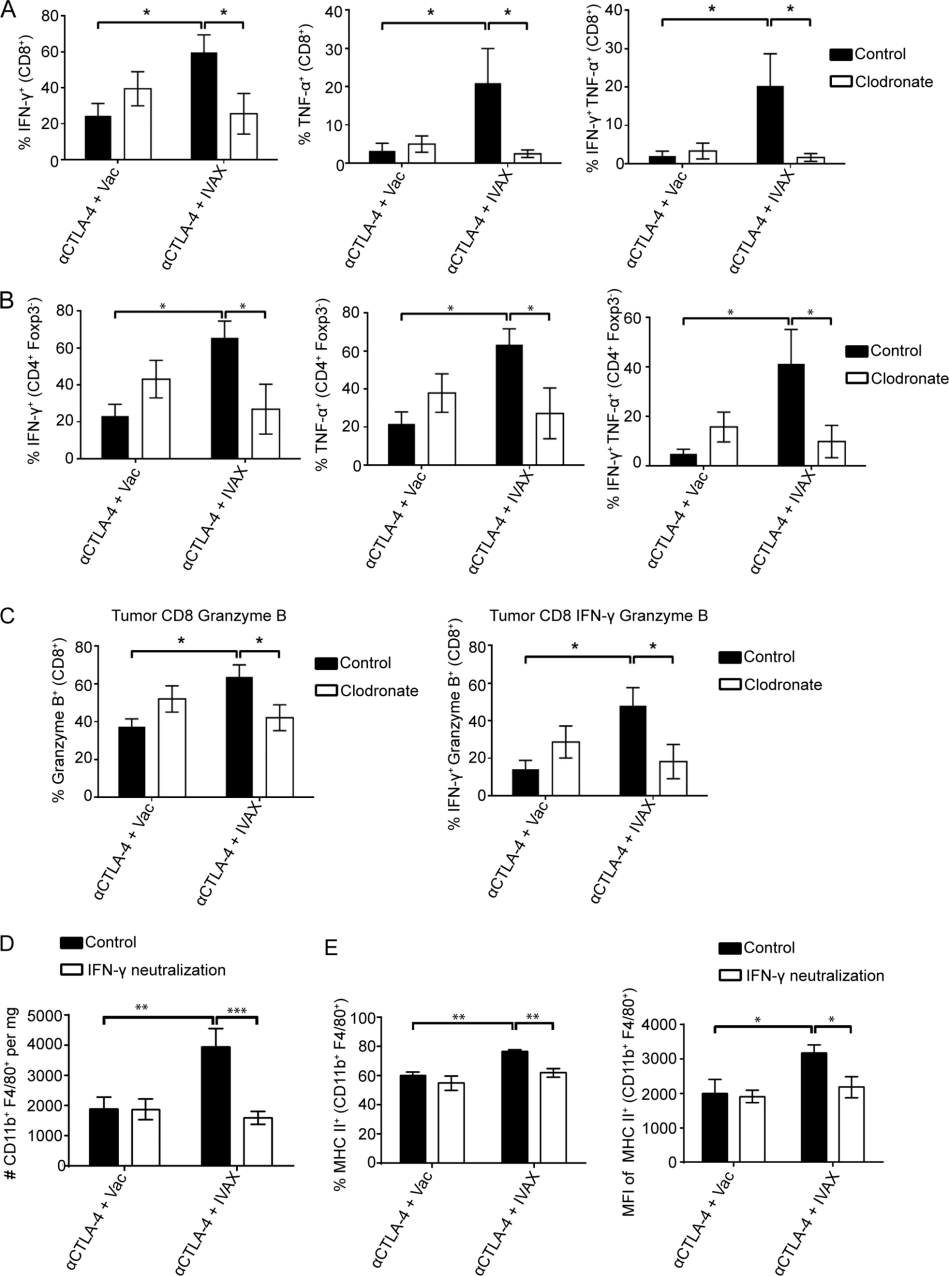
**Macrophage depletion is associated with decreased TIL functions. (A)** Cumulative frequency of tumor-infiltrating IFN-γ^+^ CD8 T cells, TNF-α^+^ CD8 T cells, and IFN-γ^+^ TNF-α^+^ CD8 T cells from two independent experiments (*n* = 3 mice per group; one-way ANOVA, post hoc, *P < 0.05). **(B)** Cumulative frequency of tumor-infiltrating IFN-γ^+^ CD4 Teff cells, TNF-α^+^ CD4 Teff cells, and IFN-γ^+^ TNF-α^+^ CD4 Teff cells from two independent experiments (*n* = 3 mice per group; one-way ANOVA, post hoc, *P < 0.05). **(C)** Cumulative frequency of tumor-infiltrating granzyme B^+^ and IFN-γ^+^ granzyme B^+^ CD8 T cells from two independent experiments (*n* = 3 mice per group; one-way ANOVA, post hoc, *P < 0.05). **(D and E)** Recruitment and polarization of M1-like macrophages depend on IFN-γ. **(D)** The density of CD11b^+^ F4/80^+^ TAMs is depicted as an absolute number of cells per mg of tumor on day 16 after tumor challenge. Data were pooled from two representative experiments out of three independent experiments (*n* = 5 mice per group; one-way ANOVA, post hoc, **P < 0.01; ***P < 0.001) **(E)** Frequency (left panel) of MHC II^+^ TAMs and mean fluorescent intensity (right panel) of MHC II molecule expressed on the surface of TAMs. Data were pooled from two representative experiments out of three independent experiments (*n* = 5 mice per group; one-way ANOVA, post hoc, *P < 0.05; **P < 0.01). Error bars represent means ± SEM. Irradiated parental B16 tumor cells (Vac) were used as a control for the IVAX group.

We next analyzed the effect of the depletion of macrophages on the T cell population. As anticipated, we found that the delayed depletion of macrophages caused profound changes in T cell populations (Figs. 6 and 7). As demonstrated previously (Fan et al., 2014) and reiterated in this study, combination therapy of IVAX and CTLA-4 blockade dramatically increases the density and frequencies of CD8 T cells and CD4^+^ Foxp3^− ^effector T cells (CD4 Teffs). However, this robust T cell immunity induced by the combination therapy was significantly dampened after delayed clodronate treatment, resulting in a roughly 50% reduction in both CD8 T cells and CD4 Teffs (Fig. 6 A). The density of Tregs in the tumor remained unaffected by either the therapy or macrophage depletion (Fig. 6 B), leading to a halving of the CD8 T cell to Treg ratio, with a lesser non-significant reduction in the CD4 Teff to Treg ratio (Fig. 6 C).

The decline in the number of antitumor T cells was accompanied by a significant decrease in their immune functions. Combination therapy induced a several-fold increase in the secretion of proinflammatory cytokines IFN-γ and TNF-α in CD8 T cells and CD4 Teffs compared with anti-CTLA-4 alone (Fig. 7, A and B). However, this robust immune activation was almost completely abrogated after macrophage depletion. The percentage of CD8 T cells and CD4 Teffs producing either IFN-γ or TNF-α was reduced to levels comparable with tumors treated with CTLA-4 blockade alone, notably losing the IFN-γ and TNF-α double-producing population. Particularly for CD8 T cells, the expression of granzyme B, a major effector molecule for direct tumor killing, was also decreased with macrophage depletion (Fig. 7 C). Overall, the diminished infiltration of CD8 T cells and CD4 Teffs into the tumor, coupled with a significant decline in their antitumor functionality and macrophage depletion, caused a broad shift in the landscape of tumor-infiltrating immune cells, potentially explaining the observed loss of tumor protection efficacy.

### The effect of macrophage depletion can be mimicked by IFN-γ blockade

IFN-γ, a paradigmatic Th1 cytokine, is a significant driver of the M1 or classical activation of macrophages (Sica and Mantovani, 2012). Given the abundance of IFN-γ produced by CD8 T cells and CD4 Teffs in tumors treated with IVAX and CTLA-4 blockade, we hypothesized that IFN-γ might play a pivotal role in a feedback loop between the T cell compartment and the macrophages in the TME. Tumor-bearing mice received an IFN-γ–neutralizing antibody in addition to combination therapy of IVAX and CTLA-4 blockade. Blocking IFN-γ closely mimicked the effect of delayed clodronate treatment, reducing both the total number of macrophages (Fig. 7 D) in the tumor and the frequency of MHC class II–expressing macrophages, along with their expression levels on those macrophages (Fig. 7 E). These results demonstrate that IFN-γ produced in the highly inflammatory TME promotes macrophage infiltration and M1 polarization, supporting our hypothesis of a positive feedback loop that IFN-γ produced by the T cells induces more M1 macrophages, which in turn helps to sustain T cell–mediated antitumor immunity.

### Macrophages also play an essential role in CTLA-4 and PD-1 dual blockade

**Figure 8.**
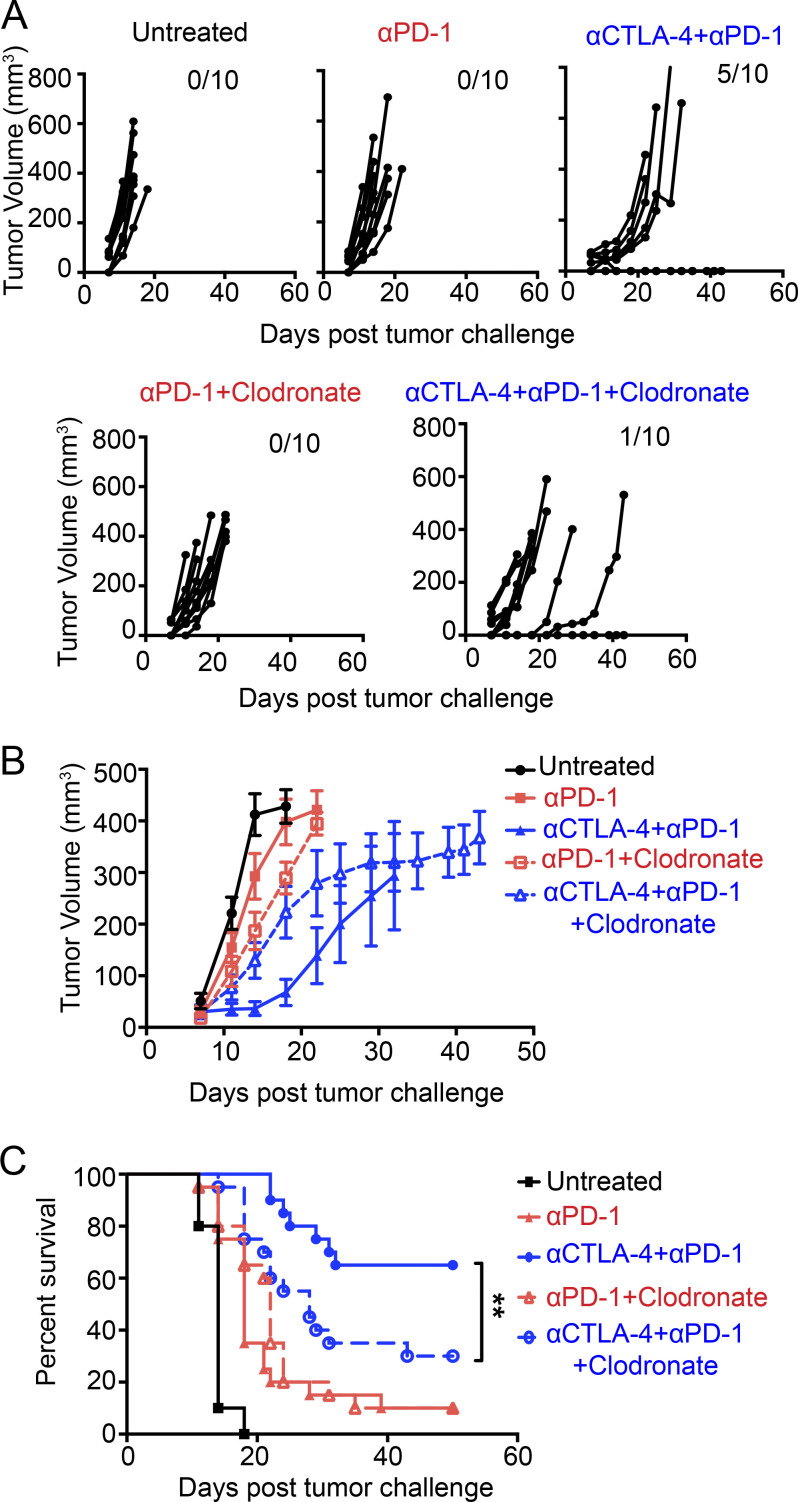
**The therapeutic efficacy of dual CTLA-4 and PD-1 blockade is also diminished by macrophage depletion.** Mice received i.d. challenges on the right flank with 2 × 10^5^ B16-F10 tumor cells. Subsequently, i.p. injections of 100 µg anti-CTLA-4 and 250 µg anti-PD-1, either individually or in combination, were administered on days 3, 6, 9, and 12 after tumor challenge. The clodronate liposomes were injected on day 7 and day 14. **(A)** Individual tumor growth curves after B16-F10 cell challenge, with upper right numbers indicating tumor-free mice. Representative data from three independent experiments (*n* = 10 mice per group). **(B)** Tumor growth curves illustrate the average tumor volume in each group. Error bars represent means ± SEM. Data are representative of three independent experiments (*n* = 10 mice per group). **(C)** Representative survival curves from three independent experiments (*n* = 10 mice per group) were analyzed using the log-rank test. **P < 0.01.

The differential impact of macrophage depletion on CTLA-4 blockade monotherapy and IVAX plus anti-CTLA-4 combination led us to hypothesize that the role of macrophages depends on the specific context within the TME. In many preclinical models and immune desert human cancers, macrophages tend to skew toward an M2-like phenotype influenced by tumor cell products (Mantovani et al., 2008) and/or other immune cell types (Biswas and Mantovani, 2010). However, there are reports of the tumor milieu favoring an M1 polarization in certain conditions, contributing to tumor rejection (Guiducci et al., 2005; Klug et al., 2013; Singh et al., 2014; Sinha et al., 2005). Besides IVAX and CTLA-4 blockade combination therapy, the combination of CTLA-4 and PD-1 blockade also elicits a potent T cell–mediated antitumor response in both mice and humans (Curran et al., 2010; Larkin et al., 2015). We investigated whether TAMs also play an essential role in the immunity generated by the anti-CTLA-4 and anti-PD-1 combination. Mice treated with PD-1 and CTLA-4 dual blockade successfully rejected the majority of B16-F10 tumors, but the therapeutic efficacy was compromised in mice subjected to delayed macrophage depletion, resulting in a survival rate drop by more than half (Fig. 8 C). The combinatorial blockade of PD-1 and CTLA-4 failed to control tumor growth in the group treated with clodronate liposome (Fig. 8, A and B). Consequently, our data support the notion that TAMs play a positive role in the antitumor immunity generated by potent combinational checkpoint blockade. Considering this evidence, we propose a positive feedback loop between intratumoral Teff cells and the TAMs, where IFN-γ produced by the T cells polarizes the TAMs into antitumor M1-like phenotype, and the TAMs, in turn, reshape the TME.

## Discussion

TAMs constitute a significant portion of tumor-infiltrating immune cells, displaying diverse roles with both immunosuppressive M2-like phenotypes as well as antitumor M1-like phenotypes, influenced by various factors such as tumor type, microenvironmental cues, and therapy regimens. While generally assuming an immunosuppressive M2-like phenotype (Mantovani et al., 2002), evidenced by studies utilizing the macrophage depletion through blockade of CSF1/CSF1R pathway (DeNardo et al., 2011; Paulus et al., 2006; Zhu et al., 2014), TAMs exhibit significant plasticity and can polarize into M1-like phenotypes under specific conditions (Wang et al., 2010; O’Sullivan et al., 2012; Biswas and Mantovani, 2010; Mantovani et al., 2002; Guiducci et al., 2005; Saccani et al., 2006; Stout et al., 2009). The balance between these phenotypes influences the tumor’s immune response.

In the present study, we demonstrated that the TAMs’ phenotype and their contribution to antitumor immunity depend on the specific TME. Potent combination immunotherapy shifts the TAMs balance toward antitumor proinflammatory or less immunosuppressive populations, as opposed to suboptimal monotherapy. These TAMs are pivotal in the recruitment and functional activity of effector CD4 and CD8 T cells, significantly improving therapy outcomes. However, the recruitment and polarization of these proinflammatory TAMs require IFN-γ. Our findings support the existence of a positive feedback loop between T cells and TAMs, mutually enhancing their function for maximum therapeutic benefit.

These results align with findings by Gubin et al. (2018), who demonstrated that combination treatment of anti-CTLA-4 and anti-PD-1 remodels TAMs to a less immunosuppressive phenotype in the MCA sarcoma model. In our study, we show through macrophage depletion and functional T cell suppression experiments that the combination therapy of anti-CTLA-4 and IVAX overall skews the TAM population to functionally less immunosuppressive and antitumor TAMs, significantly contributing to antitumor immunity (Figs. 1 and S3). However, the intricate immune-modulating roles played by the Mon/Mac in the combination therapy necessitate further high-dimensional investigations to comprehensively understand the diverse nature of the continuum of the TAM population and their functional significance in antitumor immunity.

There are several potential mechanisms through which macrophages positively contribute to antitumor immunity. First, macrophages can directly kill tumor cells or induce antibody-dependent cell-mediated cytotoxicity (Masztalerz et al., 2003). Blockade of the PD-1/PD-L1 pathway has been shown to enhance the phagocytic function of TAMs and improve the survival of tumor-bearing mice (Gordon et al., 2017). Second, macrophages can mediate “immunogenic cell death” by sensing the release of “eat-me” signals (e.g., calreticulin, ATP, and HMGB1) and enhancing antigen-presenting capacity (Kroemer et al., 2013). Third, M1-like macrophages can create a more immunogenic TME by producing Th1 chemokines and activating endothelial cells, facilitating the recruitment of Teff cells into tumors and promoting efficient T cell–mediated tumor rejection (Klug et al., 2013). Lastly, macrophages can contribute to the depletion of Tregs bound by CTLA-4 antibodies, further enhancing antitumor immunity (Selby et al., 2013; Sharma et al., 2019; Simpson et al., 2013). In our study, M1-like antitumor macrophages induced by the combination therapy likely exert their antitumor functions through a combination of these mechanisms.

The specificity of methods employed for macrophage depletion presents a persistent challenge due to the intricate nature of macrophage responses and the inherent limitations associated with the available depletion techniques. While blockade of the CSF1/CSF1R pathway has shown promise in reducing immunosuppressive M2-like macrophages (Zhu et al., 2014), recent clinical trials have yielded disappointing outcomes, emphasizing the significance of treatment timing and tumor type. Moreover, the diverse effects of CSF1/CSF1R pathway inhibition, including Treg activation, recruitment of other myeloid populations, and resistance in specific macrophage subsets, make it challenging to solely attribute observed results to macrophage depletion (Gyori et al., 2018; Kumar et al., 2017; Quail and Joyce, 2017; Zhang et al., 2020).

In our study, we opted for clodronate liposomes to selectively deplete macrophages, ensuring specific effects on this cell type. Delayed clodronate treatment moderately reduced macrophages in the spleen and lymph nodes while significantly depleting them in the tumor. Importantly, we observed no substantial impact on DCs in delayed clodronate treatment, indicating that compromised antitumor immunity primarily arises from macrophage-related factors rather than effects on the DC compartment. Additionally, our findings diverge from a recent study as we found no depletion or stunning of neutrophils in tumors, as demonstrated by the lack of changes in the surface markers expression (Culemann et al., 2023). These results underscore the specificity of clodronate liposomes in depleting macrophages in our experimental model.

Our study highlights that particular consideration needs to be given to the design of combination therapy involving macrophage depletion, especially when therapy modalities may alter the balance of immunosuppressive/antitumor macrophages in the specific TME. The potential benefit of depleting immunosuppressive macrophages should be weighed against the loss of antitumor macrophages. In situations where immunosuppressive M2-like macrophages are not the dominant subset of intratumoral myeloid cells, activation of the antitumor M1-like macrophage compartment may provide better support to the T cells, yielding improved clinical outcomes. Additionally, we demonstrate that the antitumor M1-like macrophages and antitumor Teff cells depend on each other for their infiltration and differentiation, and the positive feedback loop between them is required for optimal therapeutic efficacy of combination immunotherapy. Notably, this positive contribution to tumor rejection is particularly significant when the TME is conditioned to be more proinflammatory by potent combination immunotherapy, with IFN-γ playing a crucial role in propagating this positive feedback loop. These findings underscore the necessity of considering the specific TME and the phenotype of the TAMs when designing therapeutic approaches involving the manipulation of macrophages.

Despite a compelling clinical rationale for utilizing anti-ICOS agonists to activate the T cells, recent clinical trials have yielded disappointing results (Lee and Fong, 2022; Solinas et al., 2020). In response, we propose exploring anti-CTLA-4 antibodies, rather than anti-PD-1 antibodies, in combination with anti-ICOS antibodies in future clinical trials. This recommendation is grounded in our observation of a novel Th1 cell population expressing ICOS following CTLA-4 blockade. Previous studies by Liakou et al. (2008) identified ICOS^+^ CD4 T cells in patients with bladder cancer treated with anti-CTLA-4 antibody. We further identified a unique cluster of ICOS^+^ T-bet^+^ CD4 effector cells analogous to those identified in the study mentioned above in the murine model which developed on anti-CTLA-4 treatment or in mice where CTLA-4 was genetically deleted (Wei et al., 2017, 2019). Knocking out another checkpoint, PD-1 or anti-PD-1 treatment did not result in the appearance of these cells.

Moreover, thorough investigations are imperative to discern anti-ICOS antibodies’ agonistic/antagonistic nature as their actual properties may deviate from initial assumptions. Additionally, considering that Tregs express high levels of ICOS (Arce Vargas et al., 2018), selecting patients based on ICOS expression may not be ideal for the agonistic antibodies. Instead, patients with an induced population of ICOS^+^ CD4^+^ T cells could be the criteria for patient selection.

In summary, our study elucidates the crucial role of TAMs in the therapeutic efficacy of combination immunotherapies such as CTLA-4 blockade plus IVAX or anti-PD-1. We provide evidence that TAMs play a critical role in tumor protection and the remodeling of the TME and that certain combination therapies may remodel the TAMs to an antitumor phenotype. Therefore, macrophage depletion wouldn’t be a wise strategy in these combination therapies. Furthermore, our findings demonstrate that the combination therapy induces remodeling and activation of T cell subsets associated with antitumor immune responses, with a feedback loop mechanism between T cells and TAMs, potentially involving IFN-γ. Our study echoes Kruse et al. (2023) emphasizing Th1, IFN-γ, and macrophages in tumor eradication, showcasing a positive feedback loop between T cells and TAMs through IFN-γ, underlining the complexity of the TME. These findings offer valuable insights into the mechanisms underlying the therapeutic efficacy of IVAX and anti-CTLA-4 combination therapy. Overall, our study underscores the importance of understanding the complex interplay between TAMs and T cells in the TME, and that combining an ICOS agonist with a therapeutic combination employing CTLA-4 blockade (ipilimumab) might be valuable in increasing therapeutic efficacy.

## Materials and methods

### Mice

The study used 6–8-wk-old C57BL/6 mice obtained from the Jackson Laboratory. The obtained mice were allowed at least 1 wk for acclimatization before initiating an experiment. The mice were kept in a controlled environment free from specific pathogens, and the housing conditions were in compliance with institutional guidelines. All animal experiments were approved by the MD Anderson Cancer Center Institutional Animal Care and Use Committee.

### Cell lines and reagents

The poorly immunogenic mouse melanoma cell line B16-F10 was obtained from Dr. Isaiah Fidler (MD Anderson Cancer Center, Houston, TX, USA). The cell lines underwent authentication through spectral karyotyping to detect other cell contamination, and regular mycoplasma testing was conducted. The generation of IVAX was previously described (Fan et al., 2014). Anti-CTLA-4 (9H10), anti-PD-1 (RMP1-14), anti-Ly6-G (1A8), anti-CSF1R (AFS98), and anti-IFN-γ (XMG1.2) were purchased from BioXCell and administered intraperitoneally. Clodronate liposomes and control liposomes were purchased from https://ClodronateLiposomes.com. All the liposomes were administered i.p. at the dose recommended by the vendors. The following antibodies were used for flow cytometry analysis. Anti-CD45.2 (clone 104), anti-CD3 (clone 145-2C11), anti-CD11b (clone M1/70), anti-CD11c (clone N418), anti-F4/80 (clone BM8), anti-MHC II (I-A/I-E) (clone M5/114.15.2), anti-CD206 (clone MR5D3), anti-CD4 (clone L3T4), and anti-CD8 (53-6.7), anti-Foxp3 (clone FJK-16 s), anti-IFN-γ (clone XMG1.2), anti-TNF-α (clone MP6-XT22), anti-Ly-6G (clone 1A8), anti-Ly-6C (clone HK1.4), and anti-granzyme B (clone GB11) were purchased from eBioscience (Thermo Fisher Scientific). The functional monoclonal antibodies against mouse CD3e (clone 500A2) and CD28 (37.51) were also procured from eBioscience (Thermo Fisher Scientific). Metal-conjugated antibodies for mass cytometry were obtained from Fluidigm or unlabeled antibodies from various vendors and were conjugated with metals in-house according to the manufacturers protocol (Fluidigm) and as described earlier (Sharma et al., 2021).

### Tumor challenges and treatments

Mice were challenged i.d. on the right flank with 2 × 10^5^ B16-F10 tumor cells, which is considered day 0. In experiments where mice would be sacrificed on day 16 for functional and phenotypic analysis by flow cytometry, CyTOF, or scRNA-seq, the initial B16-F10 cells challenge was 10^6^. Mice were then treated with an i.p. injection of 100 µg anti-CTLA-4, 250 µg anti-PD-1, intradermal vaccination on the left flank with 10^6^ irradiated (150 Gy) IVAX, or 10^6^ irradiated (150 Gy) parental B16 tumor cells (Vac), used as vaccine control for IVAX, or a combination of the above on days 3, 6, 9, and 12. In the setting of early macrophage depletion, 1 mg clodronate liposome or control liposome was injected i.p. on day 0 and day 7. In contrast, the liposomes were injected on day 7 and day 14 in the delayed depletion setting. Anti-IFN-γ (200 µg) was injected i.p. on days 3, 6, 9, and 12. The mice were then followed for tumor growth or sacrificed on day 16 for the dissection of lymphoid organs and tumors. Tumor sizes were calculated from the length, width, and height measured with a digital caliper. Mice were randomly assigned to experimental groups, and the tumor measurement was conducted in a double-blinded manner. Mice that perish from causes unrelated to tumor burden are excluded from the survival graphs.

### Tumor processing, flow, and mass cytometry

Mice used for functional and phenotypic experiments were sacrificed on day 16 after the tumor challenge, and spleens, tumor-draining lymph nodes, and tumors were isolated. Tissues were digested in Liberase TL (Roche) and DNase I (Roche) at 37°C for 30 min and filtered through a 70-μm nylon cell strainer. Tumor samples for the mass cytometry or scRNA-seq analysis were cryopreserved as described earlier (Sharma et al., 2021). Both fresh or frozen tissues were centrifuged over Histopaque-1119 (Sigma-Aldrich) discontinuous gradient at 2,000 rpm for 20 min at room temperature. For functional analysis, tumor-infiltrating T cells were restimulated for 4 h at 37°C with Cell Stimulation Cocktail (eBioscience, Thermo Fisher Scientific) in the presence of Golgi-Plug (BD). These cells were first treated with Live/Dead fixable blue (Life Technologies), after which cell surface antibodies were added for staining. The cells were then fixed and permeabilized using a FoxP3 fix/perm buffer kit from eBioscience (Thermo Fisher Scientific) as per the manufacturer’s instructions and subsequently stained with intracellular antibodies to prepare for flow cytometry analysis. CountBright Absolute Counting Beads (Thermo Fisher Scientific) were added to the samples before analysis to quantify cells in the tissue. Data were acquired on BD LSR II cytometer and analyzed by FlowJo Software. For mass cytometry analysis, samples were processed, stained with mass cytometry antibodies, and analyzed using Helios mass cytometer using the Helios6.5.358 acquisition software (Fluidigm) as described earlier (Sharma et al., 2021). The data were exported for further analysis, which involved t-SNE (t-distributed stochastic neighbor embedding) dimension reduction. The Cyt tool in MATLAB software was used to perform PhenoGraph clustering analyses.

### In vitro suppression assays

Mice were challenged, and tumor-infiltrating lymphocytes (TILs) from tumors were isolated as described above. TILs were stained with anti-CD45.2, anti-CD11b, and anti-F4/80 antibodies to purify CD11b^+^ F4/80^+^ TAMs by flow sorting. 5–7 × 10^4^ WT conventional naïve T cells were incubated with TAMs isolated from different treatment groups. These cells were stimulated in vitro in a 96-well round bottom plate using anti-CD3 (1.25 µg/ml) and anti-CD28 (1.25 µg/ml) antibodies for 48 h. Monensin (0.5 μl/ml) and brefeldin A (0.5 μl/ml) (BD Biosciences) were added during the final 4 h of stimulation. After stimulation, the cells were stained with surface markers, fixed, and permeabilized using the FoxP3 Fix/Perm buffer kit (eBioscience) as per the manufacturer’s instructions. The cells were then stained with antibodies for intracellular proteins for further analysis using flow cytometry.

### scRNA-seq library generation and data processing

Histopaque-1119 purified single-cell suspensions of cryopreserved tumor tissues were stained with anti-CD45.2 antibodies and Live/Dead fixable blue (Life Technologies). The live CD45.2^+^ TILs were sorted using FACS and then encapsulated in droplets. Using the Chromium Single Cell 3′ Reagent Kits, v3 libraries were prepared as per the manufacturers protocol (10X Genomics) and as described earlier (Sharma et al., 2021), and then sequenced on an Illumina Novaseq 6000. Raw reads were aligned to the mm10 mouse reference genome and quantified using the cellranger count (v3.1.0). The individual count matrices were merged using the cellranger aggr pipeline, and detailed summary statistics can be found in Table S1. The datasets were analyzed using the R package Seurat (v3.0.0), as described earlier (Sharma et al., 2021). Briefly, genes detected in less than three cells and cells with less than 200 genes detected were filtered out. Low-quality cells with more than 10% of the transcripts derived from mitochondria genes were removed from downstream analysis. A high-quality gene cell matrix was then normalized, highly variable genes were detected, and unsupervised cell clustering was performed. Uniform Manifold Approximation and Projection (UMAP) was used for visualization (McInnes et al., 2018, *Preprint*). Only *ptprc* (CD45)-positive clusters were employed to create the graphs.

### Single-cell gene set enrichment analysis (GSEA) analysis

Single-cell GSEA was performed using the escape R package (v1.2.0), which returns the enrichment score for each cell. Cancer hallmark and KEGG pathway gene sets were derived from the Molecular Signature Database (https://www.gsea-msigdb.org/gsea/msigdb/). To test the significance of differential pathway activity between combination therapy and anti-CTLA-4 antibody treatment groups, we used a two-sided *t* test for enrichment scores comparing the two conditions.

### Statistical analyses

FlowJo (FlowJo, LLC) and Prism 7.0 (GraphPad Software, Inc.) were used to analyze the data. The experiments were conducted two to three times, and statistical significance was evaluated using multiple *t* tests, one-way ANOVA, and Bonferroni’s multiple comparisons tests. The Kaplan–Meier method was used to analyze tumor survival data, and the log-rank test was used for univariate analyses to compare survival curves for different groups. A P value of <0.05 was deemed statistically significant.

### Online supplemental material

Fig. S1 shows that a combination of IVAX and CTLA-4 blockade increases macrophage infiltration. Fig. S2 shows that reduced intratumoral macrophages correlate with loss of tumor protection. Fig. S3 shows that combination therapy reduces the suppressive efficacies of TAMs. Fig. S4 shows scRNA-seq analysis of changes in immune cell composition in tumors of different treatment groups. Fig. S5 shows a single-cell analysis of metabolic pathway activity of tumor-infiltrating immune cells in combination therapy compared with anti-CTLA-4 antibody. Table S1 shows a summary of statistics and quality control of alignment from CellRanger.

## Supplementary Material

Table S1 shows a summary of statistics and quality control of alignment from CellRanger.

## Data Availability

All scRNA-seq datasets are available in the NCBI Sequence Read Archive BioProject database under accession no. PRJNA956978. Materials generated in the course of this work may be obtained through a material transfer agreement.
