# Supplementary material for: ICOS costimulation in combination with CTLA-4 blockade remodels tumor-associated macrophages toward an antitumor phenotype
Source: J Exp Med. 2024 Mar 22;221(4):e20231263. doi: 10.1084/jem.20231263 (PMC10959121; doi:10.1084/jem.20231263)
Supplement: Table S1 — shows a summary of statistics and quality control of alignment from CellRanger. [file JEM_20231263_TableS1.docx]

**Table S1.** Summary of statistics and quality control of alignment from CellRanger.

| **Parameters** | **Vac Control** | **ɑCTLA-4 + Vac** | **IVAX** | **ɑCTLA-4 + IVAX** |
| --- | --- | --- | --- | --- |
| Estimated # of Cells | 3,806 | 5,348 | 4,358 | 4,160 |
| Fraction Reads in Cells | 89.3% | 87.7% | 90.2% | 89.0% |
| Mean Reads per Cell | 110,467 | 68,111 | 80,694 | 117,861 |
| Median Genes per Cell | 1,958 | 2,187 | 1,941 | 1,958 |
| Total Genes Detected | 17,978 | 17,826 | 18,156 | 17,414 |
| Median UMI Counts per Cell | 6,570 | 8,092 | 6,866 | 6,446 |
| Valid Barcodes | 96.9% | 97.1% | 97.3% | 96.6% |
| Reads Mapped Confidently to Transcriptome | 59.9% | 66.2% | 65.8% | 57.0% |
| Reads Mapped Confidently to Exonic Regions | 63.3% | 69.2% | 68.8% | 60.3% |
| Sequencing Saturation | 81.0% | 71.8% | 74.6% | 85.1% |
